# Vulnerable newborn phenotypes in Peru: a population-based study of 3,841,531 births at national and subnational levels from 2012 to 2021

**DOI:** 10.1016/j.lana.2024.100695

**Published:** 2024-02-15

**Authors:** Kim N. Cajachagua-Torres, Hugo G. Quezada-Pinedo, Wilmer Cristobal Guzman-Vilca, Carla Tarazona-Meza, Rodrigo M. Carrillo-Larco, Luis Huicho

**Affiliations:** a The Generation R Study Group, Erasmus MC, University Medical Center Rotterdam, Rotterdam, the Netherlands; b Department of Paediatrics, Erasmus MC, University Medical Center Rotterdam, Rotterdam, the Netherlands; c Department of Pediatrics, New York University Grossman of Medicine, New York University, New York, NY, USA; d Centro de Investigación en Salud Materna e Infantil and Centro de Investigación para el Desarrollo Integral y Sostenible, Universidad Peruana Cayetano Heredia, Lima, Peru; e Institute of Primary Health Care (BIHAM), University of Bern, Bern, Switzerland; f CRONICAS Centre of Excellence in Chronic Diseases, Universidad Peruana Cayetano Heredia, Lima, Peru; g School of Medicine “Alberto Hurtado”, Universidad Peruana Cayetano Heredia, Lima, Peru; h Sociedad Científica de Estudiantes de Medicina Cayetano Heredia (SOCEMCH), Universidad Peruana Cayetano Heredia, Lima, Peru; i Department of International Health, Bloomberg School of Public Health, Johns Hopkins University, Baltimore, MD, USA; j Nutrition and Dietetics, Universidad Científica del Sur, Lima, Perú; k Hubert Department of Global Health, Rollins School of Public Health, Emory University, Atlanta, GA, USA; l Emory Global Diabetes Research Center, Emory University, Atlanta, GA, USA

**Keywords:** Pregnancy, Birth registry, Vulnerable newborn, Maternal and child health, Developing countries

## Abstract

**Background:**

We aimed to examine the national and subnational prevalence of vulnerable newborn phenotypes in Peru, 2012–2021.

**Methods:**

Newborn phenotypes were defined using gestational age (preterm [PT], term [T]), birthweight for gestational age using INTERGROWTH-21st standards (small for gestational age [SGA], appropriate for gestational age [AGA] or large for gestational age [LGA]), and birthweight (low birthweight [LBW], non-LBW) using the Peruvian National Birth Registry as six (by excluding birthweight) and ten newborn phenotypes (using all three outcomes). Small phenotypes (with at least one classification of PT, SGA, or LBW) were further considered. Using individual-level data, we stratified the phenotypes by maternal educational level, maternal age, healthcare insurance, altitude of residence, and geographic region (Coast, Andes, and Amazon).

**Findings:**

The prevalence of the five vulnerable newborn phenotypes for the study period was LGA+T (15.2%), AGA+PT (5.2%), SGA+T (4.6%), LGA+PT (0.8%), and SGA+PT (0.7%). The Coast had a higher prevalence of newborns with large phenotypes (19.4%) and the Highlands a higher prevalence of newborns with small phenotypes (12.5%). Mothers with poor socioeconomic status, extreme ages and living at high altitude had a higher prevalence of newborns with small phenotypes, and mothers who were wealthier, more educated, and older had a higher prevalence of infants with large phenotypes.

**Interpretation:**

Our findings cautiously suggest that socioeconomic and geographic disparities may play a crucial role in shaping vulnerable newborn phenotypes at national and subnational level in Peru. Further studies using longitudinal data are needed to corroborate our findings and to identify individual-level risk factors.

**Funding:**

Ter Meulen Grant from the 10.13039/501100001722KNAW Medical Sciences Fund of the 10.13039/501100001722Royal Netherlands Academy of Arts and Sciences (KNAWWF/1085/TMB406, KNAWWF/1327/TMB202116), 10.13039/100000061Fogarty Program (D43TW011502).

**Research in context**

**Evidence before this study**

A search in PubMed was conducted on July 27th, 2023 using the following search terms: “newborn phenotypes” AND “developing countries”, with no restrictions of language or date. Out of 89 results, only one study evaluated birthweight from different country cohorts before 1998, accounting for geographical variation. This study had the objective to elucidate the effects of maternal and parental size on newborn phenotypes, where the main outcome was birthweight as well as other anthropometric measurements, without a classification into vulnerable phenotypes at birth. Furthermore, this study did not contain other national socioeconomic or contextual information. In addition to this, a recent series of studies implemented the use of six novel phenotypes of newborn globally: four small (SGA+PT, AGA+PT, LGA+PT, SGA+T), one large (LGA+T) and one reference category (AGA+T). The study examined the prevalence of vulnerable newborns from 23 countries and the time trends from 2000 to 2021. While the global prevalence remained steady, certain countries had increased trends of small phenotypes and the use of these new phenotypes, may provide precise monitoring information for countries.^1^

**Added value of this study**

From our best knowledge, this is the first study describing the vulnerable newborn phenotypes across Peru between 2012 and 2021. The analysed data from the national birth register allowed the identification of vulnerable newborn phenotypes at national, natural regions and regional levels, while exploring socioeconomic and geographical disparities. The vulnerable newborn phenotypes used in this study incorporated gestational age to previous standards, in an effort to advancing on more detailed phenotypes with differing risk. While the national prevalence of the identified newborn phenotypes did not change largely over time, differences in trends were observed for small and large phenotypes by natural region and regional levels. Importantly, the Highland (natural region) showed a higher prevalence on the small phenotypes than the Coastal and Amazonian regions. We also identified large socioeconomic disparities in the prevalence of vulnerable phenotypes.

**Implications of all the available evidence**

Our study showed a steady national vulnerable phenotypes prevalence, but significant differences between natural regions and between regions were observed. Further studies are needed to understand the potential factors driving these differences. The disparities in the vulnerable phenotypes prevalence at the subnational level need to be considered for surveillance and health interventions, which should consider designing locally-oriented policies.

## Introduction

Worldwide, every year there are 23.3 million small-for-gestational-age (SGA) newborns, 15 million preterm (PT) newborns (i.e., before 37 weeks of gestation), and 20 million low birthweight (LBW) newborns (i.e., less than 2500 g).^2^^,^^3^ SGA newborns have 83% higher risk of neonatal mortality and 90% higher risk of post-neonatal mortality than appropriate for gestational age (AGA) newborns.^4^ Therefore, SGA, PT birth and LBW are important clinical and health indicators for tracking neonatal and population health over time. SGA and PT birth have been largely associated with an increased risk of health problems in later life, including premature mortality, poor linear growth, noncommunicable disease and neurodevelopmental impairment.^5^, ^6^, ^7^, ^8^, ^9^ Although LBW have slowly reduced in the past 30 years, such decrease was not homogenous for all low- and middle-income countries (LMICs).^10^ On a global scale, we are far behind the target of a 30% reduction in the prevalence of LBW by 2030.^11^

High-income countries experienced a 3–4% decrease in PT births worldwide during the COVID-19 lockdown period, while no significant changes were observed in LMICs.^12^ Around 36% livebirths in LMICs were born either too small (i.e., SGA) or too soon (i.e., PT), or both.^4^^,^^12^ These adverse birth outcomes could predict adverse postnatal health consequences.^3^ Despite this high prevalence of SGA and PT in these countries, vulnerable newborn phenotypes have not been well described in such settings and national level studies might be hindering subnational differences. Furthermore, large for gestational age (LGA) newborns have a substantial risk of adverse short- and long-term health outcomes. Previous studies have shown that the prevalence of LGA newborns has increased over the last decade but few studies have systematically evaluated these vulnerable newborn phenotypes at subnational levels.^13^^,^^14^ Since LBW is caused by PT and/or SGA, and LGA has increased, a more comprehensive characterization of vulnerable newborn phenotypes is needed to identify infants at high risk of complications and accelerate progress toward global LBW and neonatal mortality reduction targets. Therefore, this study has adopted the level of granularity of vulnerable newborn phenotypes of the Lancet Vulnerable Newborn Series and related papers.^1^^,^^11^^,^^15^^,^^16^ To summarize pathological conditions of vulnerable newborns and provide a better understanding to stakeholders, the study considered SGA and PT identifying six phenotypes as follows: SGA+PT, AGA+PT, LGA+PT, SGA+T, AGA+T, and LGA+T.^1^ There is an imperative need for more attention to perinatal health, which are key predictors of wellbeing and quality of life.

Our study aimed to examine the national and subnational prevalence of novel vulnerable newborn phenotypes in Peru. We considered the distribution of a simplified set of six phenotypes and a set of ten non-overlapping phenotypes (including LBW) to identify patterns of vulnerability and subsequent complications.^1^ We further described the prevalence of each vulnerable newborn phenotype by time, socioeconomic and geographic characteristics.

## Methods

### Data sources

This study includes all mother–child pairs recorded in The Online Live Birth Certificate Registration System (*Sistema de Registro del Certificado de Nacido Vivo*, in Spanish) in Peru. This national birth registry system was launched in March 2012 and includes information from all healthcare providers across the country. This birth registry includes newborn information immediately after birth (sex, birthweight, and gestational age), and maternal information (age, education, and health insurance).^17^^,^^18^ This data is collected and entered in the birth registration system by health professional following standard procedures outlined in national guidelines.^17^, ^18^, ^19^ Birthweight (in grams) is collected by a trained health professional during the healthcare of the newborn, while gestational age (in weeks) is estimated based on ultrasound or last menstrual period, and confirmed with physical examination.^12^^,^^17^^,^^18^ Data can be available upon request through the Ministry of Health (https://www.minsa.gob.pe/portada/transparencia/solicitud/). This data was retrieved on March 31, 2022. The national coverage of this system has improved over the years, with 12% coverage of all projected births in Peru in 2012, 37% in 2013, 53% in 2014, 72% in 2015, 80% in 2016, 84% in 2017, 88% in 2018, 85.6% in 2019, 81.4% in 2020 and 81.5% in 2021.^17^^,^^20^ We additionally collected information on the altitude of residence area in meters above sea level (m.a.s.l.) at the province level (3rd administrative level: national > region > province) from the National institute of Statistics and Computing.^21^

### Study design and study settings

This is an ecological study at national and subnational levels in Peru. Peru is an upper-middle-income country, located in South America, with an estimated total population of 33,359,416, and a gross domestic product of US$ 223.3 billion in 2021.^22^ Peru is geographically divided in three natural regions (Coast (regions along the Pacific Ocean), Highlands (regions surrounding the Andes), and Amazon (regions in the Amazon rainforest)); and subdivided in 25 regions (equivalent to states) and 196 provinces (equivalent to counties).^21^

The main healthcare providers in Peru are SIS (*Seguro Integral de Salud*, in Spanish) run by the Ministry of Health; EsSalud (*Seguro Social de Salud*, in Spanish) run by the Ministry of Labor and covering formal employees; and private healthcare and out-of-pocket health expenditure.^23^ These healthcare provides cover ∼64%, ∼29% and ∼6% of the population; respectively.^23^ This study followed the STROBE guidelines.^24^

### Study population

We included all women-child pairs from the National Birth System between 2012 and 2021. Of 3,856,933 births, we conducted a complete-case analysis. Cleaning criteria and plausibility ranges were applied as follows leading to the exclusion of observations with: (a) missing information on birthweight (n = 1401) and birthweight <250 g or >6500 g (n = 15); (b) missing information on gestational age (n = 307) and gestational age outside the range of 22–44 weeks (n = 169); (c) missing information on multiple pregnancy (n = 4700), location (n = 652), health insurance (n = 5165), and maternal education (n = 1354); (d) maternal age younger than 9 years (n = 58); and (e) ±5 standard deviations (SD) of birthweight or gestational age (n = 1581). Finally, a total of 3,841,531 (99.6% of the initial sample size) births were included in our analysis (Supplementary Fig. 1).

We excluded provinces with <30 births (n = 1 province in the entire study period) when summarizing data at the province level. This threshold was used as a data quality control because a province is not the lowest administrative level in Peru and provinces with such low birth rates seems implausible.

### Definitions

The data was categorized based on gestational age (PT birth, up to 36 weeks or term, 37 weeks and above (T)), size for gestational age (SGA, AGA, or LGA) using the INTERGROWTH-21st international newborn size for age and sex standards (extended to include newborns from 22 to 44 weeks), and birthweight (LBW <2500 g or nonLBW ≥2500).^1^^,^^25^, ^26^, ^27^ To provide greater clarity for the terms SGA and PT, the definitions given earlier are intended to bridge the gap between the concepts and their understanding (SGA+PT, AGA+PT, LGA+PT, SGA+T, AGA+T, and LGA+T).^1^ For visualization and comparison purposes between small (SGA+PT, AGA+PT, LGA+PT, SGA+T) and large (LGA+T) phenotypes, we group all the small phenotypes in our analysis. In a secondary analysis, we expanded the list of newborn phenotypes from six to ten, including LBW: SGA+PT+LBW, AGA+PT+LBW, AGA+PT+nonLBW, LGA+PT+LBW, LGA+PT+nonLBW, SGA+T+LBW, SGA+T+nonLBW, AGA+T+LBW, AGA+T+nonLBW, and LGA+T+nonLBW.^1^ The purpose of using different phenotypes is to better discriminate vulnerability and potential mechanisms between the various combinations of conditions, rather than focusing solely on one condition (i.e., LBW).

The prevalence of each vulnerable newborn phenotype was calculated as a percentage (%) after dividing the number of births of each phenotype over the total number of live births and multiplying it by 100. We further included socioeconomic and geographic variables such as maternal education (none, kinder, complete primary, incomplete secondary, complete secondary, incomplete higher and complete higher education), maternal age (<15, 15–19, 20–24, 25–29, 30–34, 35–39, and ≥40 years old), healthcare providers (SIS, EsSalud, and private/out-of-pocket/others), and altitude of residence area in m.a.s.l. By considering these factors, the study aimed to provide a comprehensive understanding of the prevalence and potential risk factors related with vulnerable newborn phenotypes across various contexts.

### Statistical analysis

First, the prevalence of vulnerable newborn phenotypes in both the simplified model of six and the more detailed model of ten phenotypes was summarized. Second, to gain insights into the spatial patterns of vulnerable newborn phenotypes, we developed maps and trend plots to depict their prevalence across different subnational levels using *ggplot2* package. Furthermore, we performed a sensitivity analysis excluding the years before 2016 in geographic trends. Third, we examined sociodemographic and geographic disparities by disaggregating the outcomes of interest across time by maternal education, maternal age, healthcare providers and altitude of residence area. Maternal education, maternal age, healthcare provider, and altitude were evaluated as potential contributing factors to these differences. Fourth, we used equiplots to illustrate differences in vulnerable newborn phenotypes in less educated (from none to complete primary education level) and younger (at the age of 19 years or less) mothers. Our main analysis focused on the six newborn phenotypes, while the secondary analysis provides a comprehensive analysis of the ten newborn phenotypes. Finally, we examined the prevalence of small and large newborn phenotypes in the study population. All statistical analyses were performed with R version 4.1.2 (R foundation, Vienna, Austria).

### Ethics

The study was approved by the Research Ethics Committee of Universidad Peruana Cayetano Heredia, Lima, Peru (SIDISI 205540).

### Role of founding source

The funders of the study had no role in study design, data collection, formal analysis, data interpretation, or writing the manuscript. All authors had full access to the data, and collectively have final responsibility for the decision to submit for publication.

## Results

### Study population characteristics

Of all births between 2012 and 2021, 48.9% were girls, the mean gestational age was 38.7 weeks [SD 1.7] and the mean birthweight was 3263.1 g [SD 525.4]. Of all mothers, the mean maternal age was 27.9 years [SD 6.9], and 33.3% had incomplete secondary school (Table 1). From 2012 to 2021, the prevalence of small phenotypes (SGA+PT, AGA+PT, LGA+PT, and SGA+T) was 11.2%, while the prevalence of the large phenotype (LGA+T) was 15.2%. The AGA+PT (5.2%) and SGA+T (4.6%) phenotypes were the two most prevalent among small phenotypes.

**Table 1:** Characteristics of the population by year.

Year	2012	2013	2014	2015	2016	2017	2018	2019	2020	2021
**Sample size (n)**	72,484	214,270	307,418	416,620	458,863	478,562	492,273	483,389	458,827	458,825
**Newborn sex, girls (%)**	48.6	48.7	48.7	48.8	48.8	48.9	48.9	48.8	49.0	49.0
**Gestational age [median (IQR)], weeks**	39 (38–40)	39 (38–40)	39 (38–40)	39 (38–40)	39 (38–40)	39 (38–40)	39 (38–40)	39 (38–40)	39 (38–40)	39 (38–40)
**Birthweight [mean (SD)], grams**	3288 (556)	3273 (545)	3270 (531)	3260 (527)	3258 (523)	3262 (520)	3269 (522)	3262 (522)	3268 (522)	3249 (524)
**Maternal age [mean (SD)], years**	26.7 (6.9)	26.9 (6.9)	27.3 (6.9)	27.5 (6.9)	27.7 (6.9)	27.8 (6.9)	30.0 (6.9)	28.1 (6.9)	28.3 (6.9)	28.5 (6.9)
**Healthcare provider (%)**										
SIS^a^	66.4	76.2	71.4	69.4	71.2	71.3	69.6	70.1	69.2	70.8
EsSalud^b^	0.4	3.8	12.7	19.6	20.0	20.2	20.2	19.4	16.9	14.8
Private and out-of-pocket	32.7	19.4	15.2	10.5	8.2	7.8	9.1	9.5	12.5	12.8
Others	0.5	0.6	0.7	0.6	0.6	0.7	1.0	1.1	1.3	1.6
**Maternal education (%)**										
Complete higher	12.2	14.0	17.0	18.7	19.6	20.1	22.1	23.3	23.8	22.7
Incomplete higher	8.8	9.7	10.2	10.3	10.7	10.9	11.1	11.2	11.1	10.0
Complete secondary	45.6	39.8	37.2	35.8	34.6	34.5	34.4	34.5	35.3	35.2
Incomplete secondary	20.1	20.0	18.8	18.2	18.2	18.2	17.2	16.7	16.5	17.2
Complete primary	6.8	8.2	8.3	8.3	8.9	8.8	8.3	8.0	7.4	8.3
Kinder	5.9	7.3	7.5	7.5	7.0	6.5	5.9	5.5	5.2	5.7
None	0.7	1.0	1.0	1.1	1.0	1.0	0.9	0.8	0.7	0.8
**Birth place (%)**										
Health facility	99.8	99.8	99.7	99.3	99.1	98.9	99.0	99.0	98.3	97.4
Outside	0.2	0.2	0.3	0.7	0.9	1.1	1.0	0.9	1.7	2.6

a SIS: Seguro Integral de Salud in Spanish.

b EsSalud: Seguro Social de Salud in Spanish.

### Geographical trends

**Fig. 1:**
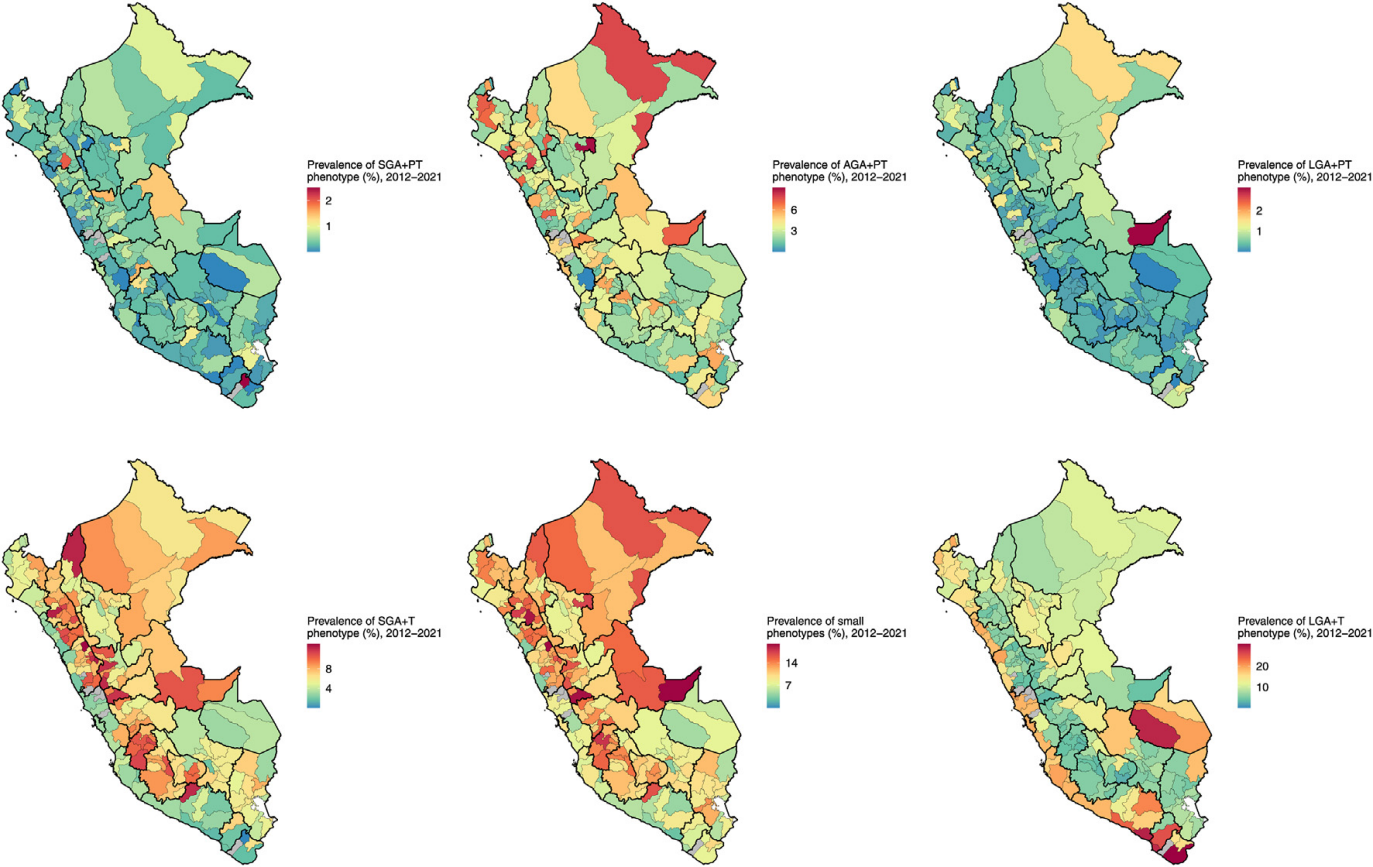
**Geographic distribution and patterns of vulnerable newborn phenotypes, Peru: 2012–2021.**^a^Small phenotypes include: SGA+PT, AGA+PT, LGA+PT, SGA+T. ^b^Large phenotype include: LGA+T. Abbreviations: Small for gestational age (SGA), preterm (PT), appropriate for gestational age (AGA), large for gestational age (LGA), and term (T).

The most prevalent simplified vulnerable newborn phenotypes across the years and per natural region were the LGA+T phenotype that had the highest prevalence in the Coast (18.9%) and the SGA+T phenotype that had the highest prevalence in the Highlands (6.5%) (Fig. 1). From 2020 to 2021, the prevalence of the LGA+T phenotype ranged from 2.1% (6/283, Grau; Highlands) to 31.6% (201/637, Islay; Coast), and the prevalence of the SGA+T phenotype ranged from 1.3% (8/637, Islay; Coast) to 16.8% (22/131, Antonio Raymondi; Highlands) (Table 2). Additionally, the prevalence of small phenotypes was the highest in the Highlands (12.5%), and the lowest in the Coast (10.3%) across the years (Fig. 1). In 2020–2021, the prevalence of small phenotype ranged from 2.5% (16/637, Islay; Coast) to 24.0 (24/100, Purus; Highlands). Furthermore, eight out of the ten provinces with the highest prevalence of SGA+PT phenotype belong to the Highlands (Table 2). In the additional analyses, no substantial differences were observed between the geographic trends from 2016 to 2021 period and the geographic trends from 2012 to 2021 (Supplementary Fig. 2).

**Table 2:** Provinces with the lowest and highest prevalence of newborn phenotypes.

		SGA+PT	AGA+PT	LGA+PT	SGA+T	AGA+T	LGA+T^b^	Small phenotypes^a^
**Coast**								
**2012–2013**	**Lowest**	Ilo 0.1 (1/668)	Zarumilla 0.4 (1/276)	Huaral 0.1 (1/801)	Viru 1.6 (4/258)	Tacna 62.5 (2461/3937)	Talara 8.8 (10/114)	Ascope 2.4 (4/165)
	**Highest**	Castilla 1.9 (1/53)	Chiclayo 9.1 (174/1912)	Chiclayo 2.4 (45/1912)	Gran Chimu 8.7 (4/46)	Talara 82.5 (94/114)	Tacna 30.0 (1180/3937)	Chiclayo 19.0 (364/1912)
**2014–2015**	**Lowest**	Ilo 0.1 (2/2365)	Contralmirante Villar 0.9 (2/228)	Ferreñafe 0.1 (1/1425)	Islay 1.1 (8/718)	Tacna 62.5 (5829/9322)	Gran Chimu 9.1 (15/164)	Islay 2.5 (18/718)
	**Highest**	Sullana 1.1 (96/8707)	Piura 8.3 (1441/17,425)	Callao 1.4 (395/28,457)	Gran Chimu 10.3 (17/164)	Zarumilla 80.4 (434/540)	Tacna 30.8 (2874/9322)	Piura 16.1 (2814/17,425)
**2016–2017**	**Lowest**	Sechura 0.1 (1/1534)	Zarumilla 0.8 (5/594)	Viru 0.1 (2/1848)	Caraveli 1.1 (3/283)	Tacna 62.1 (6273/10,094)	Gran Chimu 7.2 (15/207)	Caraveli 2.8 (8/283)
	**Highest**	Gran Chimu 1.4 (3/207)	Chiclayo 7.8 (2092/26,847)	Callao 1.6 (564/34,844)	Gran Chimu 9.7 (20/207)	Sechura 83.1 (1275/1534)	Tacna 30.2 (3050/10,094)	Sullana 15.6 (1630/10,481)
**2018–2019**	**Lowest**	Ascope 0.0 (1/2320)	Islay 0.8 (6/716)	Casma 0.1 (1/946)	Islay 0.8 (6/716)	Tacna 60.0 (5906/9840)	Gran Chimu 8.2 (22/268)	Islay 2.0 (14/716)
	**Highest**	Gran Chimu 1.1 (3/268)	Sullana 7.9 (1049/13,284)	Callao 1.6 (612/39,255)	Ferreñafe 5.8 (93/1615)	Zarumilla 83.7 (507/606)	Tacna 32.1 (3156/9840)	Sullana 13.9 (1846/13,284)
**2020–2021**	**Lowest**	Ascope 0.1 (1/1915)	Zarumilla 0.4 (4/928)	Islay 0.2 (1/637)	Islay 1.3 (8/637)	Tacna 61.5 (4907/7975)	Gran Chimu 8.3 (40/480)	Islay 2.5 (16/637)
	**Highest**	Piura 1.0 (235/24,351)	Chiclayo 7.8 (1736/22,341)	Callao 1.8 (617/33,353)	Gran Chimu 6.7 (32/480)	Zarumilla 83.0 (770/928)	Islay 31.6 (201/637)	Chiclayo 14.1 (3159/22,341)
**Highlands**								
**2012–2013**	**Lowest**	Huanta 0.1 (1/943)	Pachitea 0.9 (2/223)	Andahuaylas 0.1 (1/1221)	Caylloma 2.4 (7/290)	Cajamarca 69.9 (3623/5180)	Sucre 1.6 (1/63)	Caylloma 4.1 (12/290)
	**Highest**	Cajamarca 2.4 (125/5180)	Cajamarca 9.4 (489/5180)	Ayabaca 1.6 (4/250)	Vilcas Huaman 16.1 (24/149)	Urubamba 88.0 (88/100)	Caylloma 17.2 (50/290)	Cajamarca 24.7 (1277/5180)
**2014–2015**	**Lowest**	Lucanas 0.1 (1/950)	El Collao 0.7 (9/1265)	Tayacaja 0.1 (1/1868)	Condesuyos 1.7 (1/59)	Bolivar 70.4 (38/54)	Recuay 1.3 (3/231)	Condesuyos 1.7 (1/59)
	**Highest**	Chumbivilcas 2.4 (2/82)	Cajamarca 7.8 (887/11,398)	Bolivar 3.7 (2/54)	Julcan 16.5 (23/139)	General Sanchez Cerro 94.6 (35/37)	Sandia 21.3 (60/282)	Sihuas 25.7 (54/210)
**2016–2017**	**Lowest**	Huanta 0.1 (2/3248)	Antonio Raymondi 0.7 (1/136)	Tayacaja 0.1 (1/1892)	Condesuyos 0.7 (1/141)	La Union 68.6 (24/35)	Sucre 0.8 (1/125)	Condesuyos 2.8 (4/141)
	**Highest**	La Union 5.7 (2/35)	Chachapoyas 7.3 (245/3351)	Pataz 1.6 (19/1165)	Lauricocha 14.5 (59/406)	Victor Fajardo 91.1 (102/112)	Caylloma 22.0 (574/2608)	La Union 22.9 (8/35)
**2018–2019**	**Lowest**	Huanta 0.1 (2/3174)	Mariscal Luzuriaga 0.7 (1/148)	Tayacaja 0.0 (1/2106)	Paruro 1.3 (1/75)	Caylloma 71.8 (1940/2702)	Huaytara 1.3 (2/153)	Moho 3.9 (5/129)
	**Highest**	Marañon 2.0 (6/303)	Cajamarca 8.2 (1204/14,755)	Tarata 3.3 (1/30)	San Miguel 15.8 (48/304)	Bolivar 90.1 (145/161)	Caylloma 24.1 (652/2702)	San Miguel 20.4 (62/304)
**2020–2021**	**Lowest**	Cotabambas 0.1 (1/1759)	Acomayo 0.4 (1/237)	Pachitea 0.0 (1/2143)	Moho 2.1 (4/187)	Caylloma 72.7 (2130/2931)	Grau 2.1 (6/283)	Paruro 3.1 (5/160)
	**Highest**	Cajamarca 1.9 (262/14,029)	Chachapoyas 8.3 (259/3129)	San Miguel 1.9 (8/413)	Antonio Raymondi 16.8 (22/131)	Paruro 93.8 (150/160)	Caylloma 22.2 (652/2931)	Sihuas 22.3 (124/555)
**Amazon**								
**2012–2013**	**Lowest**	Utcubamba 0.2 (1/581)	Bongara 1.0 (1/105)	Tambopata 0.1 (1/1153)	Bongara 1.9 (2/105)	Condorcanqui 67.4 (29/43)	Rodriguez de Mendoza 6.7 (10/149)	Bongara 2.9 (3/105)
	**Highest**	Coronel Portillo 1.7 (13/755)	Condorcanqui 9.3 (4/43)	Condorcanqui 2.3 (1/43)	Condorcanqui 11.6 (5/43)	Rodriguez de Mendoza 85.9 (128/149)	Tambopata 19.9 (229/1153)	Condorcanqui 23.3 (10/43)
**2014–2015**	**Lowest**	Rioja 0.1 (1/1759)	Bongara 0.6 (2/320)	Mariscal Caceres 0.1 (1/790)	Tahuamanu 2.5 (6/237)	San Martin 70.4 (5071/7206)	Requena 3.0 (1/33)	Mariscal Caceres 6.5 (51/790)
	**Highest**	Ucayali 2.4 (1/41)	San Martin 9.7 (701/7206)	Mariscal Ramon Castilla 2.8 (10/351)	Datem del Marañon 15.6 (60/385)	Requena 84.8 (28/33)	Tambopata 19.2 (906/4728)	Atalaya 19.0 (148/777)
**2016–2017**	**Lowest**	Mariscal Caceres 0.1 (1/1947)	Tahuamanu 0.8 (2/241)	San Ignacio 0.2 (3/1654)	Manu 1.3 (2/160)	Manu 64.4 (103/160)	Purus 4.4 (4/90)	Tahuamanu 5.0 (12/241)
	**Highest**	Coronel Portillo 1.5 (303/20,310)	San Martin 8.9 (914/10,264)	Huallaga 2.2 (8/367)	Condorcanqui 12.6 (107/852)	Bongara 85.3 (355/416)	Manu 30.6 (49/160)	San Martin 16.6 (1701/10,264)
**2018–2019**	**Lowest**	Mariscal Ramon Castilla 0.1 (1/1913)	Manu 0.5 (1/189)	San Ignacio 0.2 (3/1545)	Picota 3.0 (24/808)	Manu 67.7 (128/189)	Putumayo 4.1 (4/98)	Manu 4.2 (8/189)
	**Highest**	Coronel Portillo 1.4 (289/20,927)	San Martin 8.7 (931/10,668)	Purus 2.9 (3/103)	Condorcanqui 13.0 (104/801)	Putumayo 86.7 (85/98)	Manu 28.0 (53/189)	Purus 19.4 (20/103)
**2020–2021**	**Lowest**	Puerto Inca 0.1 (1/1088)	Bongara 1.0 (4/385)	Datem del Marañon 0.1 (1/1297)	Huallaga 2.7 (11/414)	Manu 66.7 (182/273)	Purus 3.0 (3/100)	Manu 5.1 (14/273)
	**Highest**	Datem del Marañon 1.4 (18/1297)	Purus 9.0 (9/100)	Purus 5.0 (5/100)	Putumayo 12.3 (20/163)	Bongara 85.2 (328/385)	Manu 28.2 (77/273)	Purus 24 (24/100)

Results are presented as prevalence estimate in percentage.

Abbreviations: Small for gestational age (SGA), preterm (PT), appropriate for gestational age (AGA), large for gestational age (LGA), and term (T).

a Small phenotypes include: SGA+PT, AGA+PT, LGA+PT, SGA+T.

b Large phenotype includes: LGA+T.

When grouping by ten vulnerable newborn phenotypes, the most common phenotype, specially across Coastal regions, was the LGA+T+NBW, which ranged from 2.6% (8/304, Antabamba, Highlands) to 30.7% (12,637/41,168, Tacna; Coast). Each of the remaining phenotypes represented less than 12% of all births in all provinces. Regarding the SGA+PT+LBW phenotype, eight out of the ten provinces with the highest prevalence were in the Highlands (Supplementary Fig. 3).

### Time trends

**Fig. 2:**
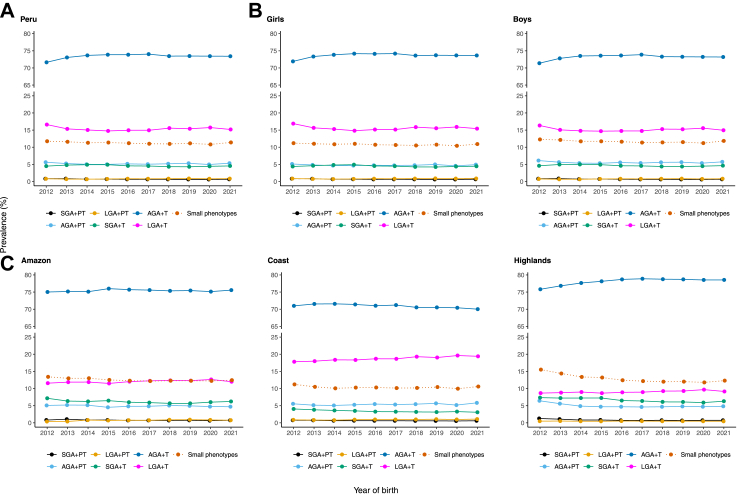
**Temporal trends of six newborn phenotypes by national (A), by sex (B) and by natural regions (C), 2012–2021.**^a^Small phenotypes include: SGA+PT, AGA+PT, LGA+PT, SGA+T. ^b^Large phenotype include: LGA+T. Abbreviations: Small for gestational age (SGA), preterm (PT), appropriate for gestational age (AGA), large for gestational age (LGA), and term (T).

The prevalence of the five vulnerable newborn phenotypes was 15.2% (585,806/3,841,531) for LGA+T, 5.2% (197,943/3,841,531) for AGA+PT, 4.6% (175,143/3,841,531) for SGA+T, 0.8% (30,788/3,841,531) for LGA+PT, and 0.7% (25,549/3,841,531) for SGA+PT across the entire study period. The most prevalent phenotype was AGA+T (the reference group), accounting for 73.6% (2,826,302/3,841,531) of all cases. The prevalence of vulnerable newborn phenotypes remained steady, with minor changes for AGA+T and LGA+T. The AGA+T phenotype increased by 1.8% (from 71.6% [51,927/72,484] to 73.4% [336,824/458,825]) over time, whereas the LGA+T phenotype decreased by 1.4% (from 16.6% [12,039/72,484] to 15.2% [69,687/458,825]). The prevalence of other vulnerable newborn phenotypes changed by less than 1%. When all small phenotypes were combined, their prevalence remained lower than that of the large phenotype. This difference remained throughout the study period (Fig. 2A). A similar pattern in the prevalence of small and large phenotypes was observed by sex, respectively (Fig. 2B).

All natural regions showed a small but steady increase in LGA+T over time. In the Highlands, the AGA+PT decreased by 1.6% (from 6.4% [521/8101] to 4.8% [6155/127,557]), SGA+T decreased by 1.0% (from 7.3% [595/8101] to 6.3% [8038/127,557]), and AGA+T increased by 2.8% (from 75.8% [6142/8101] to 78.6% [100,197/127,557]) between 2012 and 2021. The prevalence of other vulnerable newborn phenotypes varied slightly by natural region, with changes of less than 1%. In 2021, the Coast showed a substantially higher prevalence of LGA+T than the Amazon and the Highlands (19.4% [47,943/247,163], 12.0% [10,076/84,039], and 9.1% [11,663/127,557], respectively). Differences were observed across natural regions when comparing small and large phenotypes. While the Amazon had a similar prevalence of small and large phenotypes, the Coast had a higher prevalence of large than small phenotypes, and the Highlands had a higher prevalence of small than large phenotypes (Fig. 2C). Additionally, slight differences in the trends were found between the pre-pandemic and COVID-19 pandemic periods. At the national level, in both sexes, and in the Highlands, small phenotypes displayed a positive slope during the pandemic period, while the large phenotype showed a negative slope (Fig. 2A–C).

At the subnational level, most regions showed a negative trend in AGA+PT and a positive trend in LGA+T over time. The largest drops in AGA+PT were observed in Cajamarca (5.9%, from 10.7% [125/1172] to 4.8% [996/20,740]), Madre de Dios (5.2%, from 8.2% [6/73] to 3.0% [113/3769]) and San Martin (3.4%, from 8.9% [9/101] to 5.5% [901/16,393]) between 2012 and 2021. The largest increases in LGA+T were observed in San Martin (5.3%, from 7.9% [8/101] to 13.2% [2159/16,393]), Moquegua (4.2%, from 25.2% [345/1368] to 29.4% [548/1863]) and Callao (3.6%, from 16.9% [1172/6945] to 20.5% [3227/15,704]) between 2012 and 2021. In 2021, the prevalence of LGA+T was more than 10% in 17 out of the 25 regions. When small and large phenotypes were grouped, their prevalence varied greatly. The small phenotypes were more prevalent in Ayacucho, Cajamarca, Huancavelica, Junin, Loreto, Pasco, Puno, and Ucayali, whereas the large phenotype was more prevalent in Arequipa, Callao, Ica, La Libertad, Lambayeque, Lima, Madre de Dios, Moquegua, Tacna, and Tumbes. The prevalence of small and large phenotypes was similar in Amazonas, Ancash, Apurimac, Cusco, Huanuco, Piura and San Martin (Supplementary Fig. 4). No substantial changes were observed in the other vulnerable newborn phenotypes (Supplementary Fig. 4). The trends remained consistently among both girls and boys (Supplementary Figs. 5 and 6). Furthermore, the analysis of the ten vulnerable newborn phenotypes showed similar trends in small and large phenotypes (Supplementary Figs. 7–10).

### Socioeconomic and geographic inequalities (ecological analysis)

**Fig. 3:**
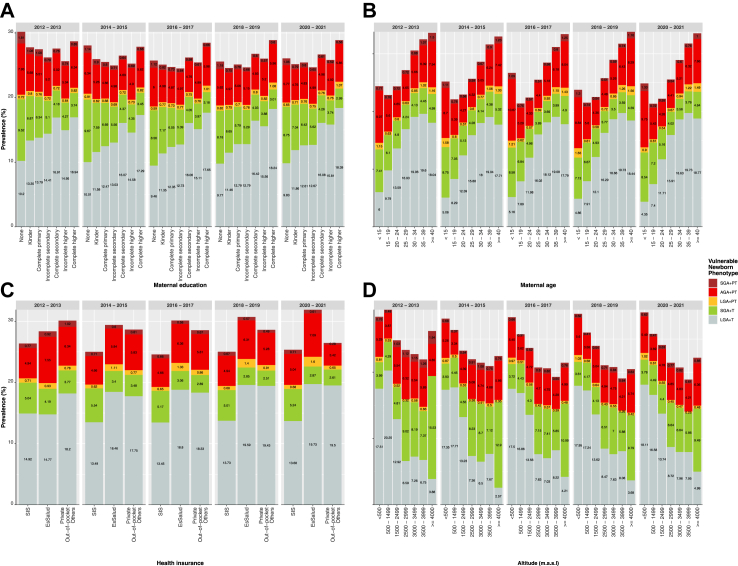
**Socioeconomic and geographic disparities of vulnerable newborn phenotypes in terms of (A) maternal education, (B) maternal age, (C) healthcare provider, and (D) altitude of residence, Peru: 2012–2021.** Abbreviations: Small for gestational age (SGA), preterm (PT), appropriate for gestational age (AGA), large for gestational age (LGA), and term (T). SIS (Seguro Integral de Salud, in Spanish) is the main healthcare provider in Peru overseen by the Ministry of Health, following by EsSalud (Seguro Social de Salud, in Spanish) run by the Ministry of Labor, covering formal employees and their families.

We examined sociodemographic disparities by disaggregating the outcomes of interest across time by maternal education, maternal age, healthcare providers and altitude of residence area. Small phenotypes were more common in less educated and/or younger mothers, whilst the large phenotype was more common in mothers with higher education (Fig. 3). Consistently, over time, the prevalence of newborns with the SGA+T phenotype was higher among less educated mothers, followed by the SGA+PT phenotype; conversely, the LGA+T and LGA+PT phenotypes increased among more educated mothers (Fig. 3A). The SGA+T phenotype was more common in newborns born from younger mothers (Fig. 3B), while the AGA+PT phenotype was more common among mothers at both extreme of age and education (Fig. 3A and B). The prevalence of newborns with the LGA+T phenotype increased with the maternal age (Fig. 3B). Over time, the prevalence of newborns with the SGA+T phenotype was consistently higher in younger mothers with complete primary or lower education (Supplementary Fig. 11). Newborns with SGA+T phenotype were more common among SIS healthcare beneficiaries, whereas newborns with the LGA+T, AGA+PT and LGA+PT phenotypes were more common among the EsSalud healthcare beneficiaries (Fig. 3C). Finally, small phenotypes (mainly, SGA+T phenotype) were more prevalent at high altitude, whereas the LGA+T phenotype was more prevalent at lower altitude (Fig. 3D). The analysis of the ten vulnerable newborn phenotypes showed similar patterns (Supplementary Figs. 12–15).

## Discussion

### Main findings

This study provides information on the newborn vulnerable phenotypes according to geographic settings in Peru for a decade period. The prevalence of newborns with large and small phenotypes were 15.2% and 11.2%, respectively. Both phenotypes exhibited minor variation at national level over time. However, the prevalence of vulnerable newborn phenotypes varied significantly at the subnational level. The Coast had a higher prevalence of newborns with large phenotypes than small phenotypes, while the Highlands and the Amazon (natural regions) had higher prevalence of newborns with small phenotypes than large phenotypes. Overall, mothers with poor socioeconomic status and living at high altitude had a higher prevalence of newborns with small phenotypes, while mothers who were apparently wealthier had a higher prevalence of infants with large phenotypes. Our findings suggest that geographic and socioeconomic disparities may play a crucial role in shaping distribution of vulnerable newborn phenotypes at national and subnational level in Peru.

### Strengths and limitations

Strengths of this study include using a national registry of births collated over ten years, which allowed the analysis of vulnerable newborn phenotypes at both the national and subnational levels. The inclusion of births from both public and private healthcare facilities across the country enabled the examination of disparities and changes by different geographical, socioeconomic, and healthcare access profiles. Furthermore, the use of six and ten newborn phenotypes might improve the identification of vulnerability, paving the way to a more comprehensive understanding of the underlying biological mechanisms and facilitating the design of targeted interventions and policies to effectively reduce vulnerability at the subnational levels.^1^ Thus, this study provides valuable findings that may assist national and international stakeholders in enhancing maternal and child health in Peru, especially in disadvantaged settings.

Some limitations must be addressed. Firstly, although the information was registered by health professionals, misclassification bias may be possible due to differences in measurement and registration procedures across medical centres and provinces. However, the overall geographic patterns and time trends should not be substantially modified, as data entry in the national registration system followed standard procedures and was carried out by qualified health professionals in accordance with national guidelines.^17^^,^^18^ Secondly, some births may have been missing from the online registration form, i.e., births in rural facilities or in those areas with poor internet connection. This might have affected our findings, particularly in the early years of the study period during the implementation of the birth registry system (2012–2014) and might have resulted in the underestimation of the vulnerable phenotypes. Although we also noticed a slight decrease in the recorded births between 2019 and 2021 compared to 2018, the coverage information consistently remained above 81% throughout these years. Thus, our findings for the earlier years (2012–2014), including any inferences regarding the prevalence of vulnerable newborn phenotypes, should be interpreted with caution for the entire population. Additionally, since there is no data available on neonatal mortality, the prevalence of vulnerable newborn might be underestimated. However, research tracking health indicators using solely live births at the state or county level have contributed to the global body of evidence.^28^, ^29^, ^30^ Third, information on pregnancy complications and lifestyle factors (e.g., body mass index, supplementation, and substance use) was not available. Further studies are needed to examine the association of these factors with vulnerable newborn phenotypes in Peru. Finally, since information on the method of assessing gestational age was absent from the birth registry, the accuracy of gestational age assessment may vary between methods. For example: first-trimester ultrasound measurements may be more accurate than the last menstrual period. Similar to births occurring in low-income settings, the Highlands and the Amazon are less likely to benefit from optimal obstetric evaluations.^1^ If the last menstrual period dating method was predominantly used in these regions, it might have contributed to an overestimation of small vulnerable phenotypes.^1^ Notably, this issue is not unique to our study and has been identified in several regions such as Asia, North Africa, and Latin America.^1^ However, despite these challenges, studies assessing health indicators in these areas still provided valuable insights.^1^ This study highlights the need to update the birth registration system and include information about the gestational age assessment method.

### Potential explanations

An accurate definition of vulnerable newborn is critical to improve targeted clinical care, since infants born with any vulnerable phenotype have a higher risk of death in the first month of life, of neurodevelopmental impairment, or a higher risk of developing noncommunicable diseases in adulthood.^5^, ^6^, ^7^, ^8^, ^9^^,^^11^^,^^16^^,^^31^ Although LBW is a well-established predictor of newborn vulnerability, PT and SGA are further key predictors of short- and long-term pathological conditions over time.^11^^,^^13^^,^^14^^,^^16^^,^^32^^,^^33^ Furthermore, LGA must also be considered a category of vulnerable newborn, since LGA newborns have a higher risk of negative health outcomes in childhood.^13^^,^^14^^,^^32^ Thus, PT, SGA and LGA may further represent the driving pathways for vulnerability, guiding the prioritization of preventive interventions and clinical care.^1^^,^^15^ However, even if a previous ecological research in Peru found the prevalence of newborns with LBW and SGA was 6.2% and 5.2%, respectively,^34^ no earlier study had comprehensively examined all these novel vulnerable newborn phenotypes at the subnational level in Peru.

Our study found that the large phenotype was more prevalent at the national level. However, when examining the prevalence by natural region, the Highlands and the Amazon had a higher prevalence of small babies than the Coast, whereas the Coast had a higher prevalence of large babies than the Highlands and the Amazon. Additionally, adolescent mothers with complete primary education or lower had prevalence of small babies (mostly, SGA+T and AGA+PT). Over time. Geographic and socioeconomic gaps may explain these differences between and within natural regions.^35^^,^^36^ The potential mechanisms underlying newborns’ vulnerability are multifaceted, including maternal health conditions, socioeconomic inequalities, and environmental factors.^15^ Peru has substantial socioeconomic disparities through their regions.^35^ The Highlands and the Amazon are characterized by challenging topographical and socioeconomic conditions, with a substantial concentration of people living in poverty and with a limited access to education and health services.^23^^,^^35^ Several regions in the Highlands (e.g., Ayacucho, Cajamarca, Huancavelica, Huánuco, Pasco and Puno) and in the Amazon (e.g., Loreto) belong to the poorest quintile.^35^ The Coast is characterized for a more extensive developing economy and higher access to health services, although there are still pockets of poverty and inequality, mainly in the coastal rural areas.^35^

Subnational disparities may further be related to individual characteristics such as differences in maternal socioeconomic and geographic characteristics.^4^^,^^37^^,^^38^ We found a widening gap overtime by maternal education level, age, type of health insurance and altitude of residence area. Prevalence of small babies was more common among mother who were younger, less educated, had public health insurance, and lived at high altitude, whereas the prevalence of large babies was more common among mothers who were older, more educated, older, had social health insurance, and lived at sea level. Growing evidence is linking low socioeconomic status and/or living at high altitude with small babies (SGA and PT),^4^^,^^37^, ^38^, ^39^ however, no previous studies described differences within phenotypes of small babies (mainly the SGA+T and SGA+PT phenotypes). This is a call for more research studies using a detailed categorization of phenotypes because some phenotypes may have the worst prognosis than other phenotypes.^5^, ^6^, ^7^, ^8^, ^9^^,^^11^^,^^16^^,^^31^^,^^33^ A previous meta-analysis among 1,604,770 newborns found that those born at high altitude (beyond 2500 m.a.s.l.) have a higher risk of LBW, SGA, and PT than those born at low altitude (below 2500 m.a.s.l.).^39^ These observed associations may be attributed, in part, to a reduced exchange of oxygen and nutrients between the mother and the foetus because of reduced uterine artery diameter and blood flow in high-altitude pregnancies.^39^^,^^40^ Other previous meta-analysis among 59,670,142 adolescent mothers found that those mothers with low socioeconomic status who lived in rural residence had a higher risk of preterm birth and low birthweight babies.^41^ Moreover, previous study in England among 1,155,981 mothers found that mothers with socioeconomic inequalities have an increased risk of preterm births, foetal growth restriction and stillbirths.^38^ Besides, younger women with low socioeconomic status may have an accumulation of adverse risk factors, including inadequate prenatal care, domestic violence and unhealthy lifestyle factors.^38^ To what extent the altitude-related mechanism, as well as socioeconomic and lifestyle factors contribute to the increased prevalence of small phenotypes in our current study remains unknown and falls outside the scope of this study. More research using longitudinal data is needed to examine the influence of adverse sociodemographic and geographic factors on vulnerable newborn phenotypes.

### Public health implications

Our findings provide information to improve the identification of vulnerable newborns in Peru, with detailed national and subnational trends, facilitating surveillance and targeted intervention programs to effectively reduce vulnerability. Given the significant impact of vulnerable newborn phenotypes on the risk of children's morbidity and mortality, providing a more comprehensive description of newborn phenotypes, including their socioeconomic and geographical characteristics, will play a pivotal role to effectively reduce the vulnerable disease burden among newborns and infants living in Peru. Decisionmakers and stakeholders can allocate resources and interventions more effectively and efficiently by knowing where vulnerable phenotypes are more common. Multisectoral efforts are required to reduce the prevalence of vulnerable newborns. According to the World Health Organization, every pregnant woman and newborn must receive equal opportunities during quality care and education throughout the pregnancy, childbirth and the postnatal period.^42^ This approach must include developing locally-oriented policies compassing nutritional interventions, comprehensive maternal and foetal assessment, multipronged preventive measures, psychological well-being interventions, and fostering communication among healthcare providers, pregnant women and families.^42^, ^43^, ^44^ Addressing disparities in the prevalence of vulnerable phenotypes at the subnational level is crucial component of surveillance and targeted health interventions. The targeted interventions optimize the potential to reduce neonatal mortality rate and prevent long-term disabilities in the population.

Employing ten newborn phenotypes provides a comprehensive characterization of vulnerable newborns and assists clinicians in identifying those at the highest risk.^1^ Nevertheless, adopting a more parsimonious approach by reducing the number of categories to six phenotypes gives a simpler and more practical method for routine implementation, while effectively pinpointing newborns at an elevated risk of premature mortality.^1^ Identifying locations with a high prevalence of newborns phenotypes at the highest risk of mortality is essential in Peru for informing preventive programs and policies across all levels of decision making, from local to national authorities. This information could guide the implementation of targeted public health interventions during pregnancy and the perinatal period. The current study offers a detailed overview of vulnerable newborn phenotypes in Peru, a LMIC setting, serving as a starting point for further investigation in this area. Further studies are required to assess the long-term consequences of the vulnerable newborn phenotypes and to better understand the specific impact of each newborn type.

## Conclusions

In Peru, both large and small phenotypes are prevalent, and their prevalence did not change substantially during the observation period. The large phenotype clustered in the coastal regions, whereas small phenotypes clustered in the Highlands. Women with higher economic status and better education exhibited a higher prevalence of the large phenotype, whereas women with lower economic status, lower education and living at high altitude showed a higher prevalence of small phenotypes. To effectively improve maternal and child health outcomes, it is crucial to consider these factors and tailor policies and healthcare interventions accordingly.

## Contributors

All authors conceived the research question and analysis plan. KNC-T pooled and prepared the data. KNC-T, HGQ-P and WCG-V conducted the analyses and prepared the figures. KNC-T prepared the first draft of the manuscript. KNC-T, HGQ-P, WCG-V, RMC-L, CT-M and LH provided critical scientific and editorial input to improve the manuscript. All authors approved the submitted version.

## Data sharing statement

All data analysed in this study are openly accessible and can be requested from the Ministry of Health in Peru at https://www.minsa.gob.pe/portada/transparencia/solicitud/frmFormulario.asp and from the National Institute of Statistics and Computing at http://iinei.inei.gob.pe/microdatos. We have provided the analysed data and maps as supplementary materials to this paper.

## Declaration of interests

None.
