# Supplementary material for: Vulnerable newborn phenotypes in Peru: a population-based study of 3,841,531 births at national and subnational levels from 2012 to 2021
Source: Lancet Reg Health Am. 2024 Feb 15;31:100695. doi: 10.1016/j.lana.2024.100695 (PMC10945436; doi:10.1016/j.lana.2024.100695)
Supplement: Supplementary Figures S1–S15 and Supplementary Table S1 [file mmc1.pdf]

Supplementary material

Vulnerable Newborn Phenotypes in Peru: A population-based study of 3,841,531 Births at National and Subnational Levels from 2012 to 2021

Kim N Cajachagua-Torres MSc[1,2,3, 4]

Hugo G Quezada-Pinedo PhD[1,2,4,5]

Wilmer Cristobal Guzman-Vilca MD[6,7,8]

Carla Tarazona-Meza MPH[9,10]

Rodrigo M Carrillo-Larco PhD[11,12]

Luis Huicho MD[4,7]

1 The Generation R Study Group, Erasmus MC, University Medical Center Rotterdam, Rotterdam, the Netherlands

2 Department of Paediatrics, Erasmus MC, University Medical Center Rotterdam, Rotterdam, the Netherlands

3 Department of Pediatrics, New York University Grossman of Medicine, New York University, New York, USA

4 Centro de Investigación en Salud Materna e Infantil and Centro de Investigación para el Desarrollo Integral y Sostenible, Universidad Peruana Cayetano Heredia, Lima, Peru

5 Institute of Primary Health Care (BIHAM), University of Bern, Bern, Switzerland

6 CRONICAS Centre of Excellence in Chronic Diseases, Universidad Peruana Cayetano Heredia, Lima, Peru

7 School of Medicine "Alberto Hurtado", Universidad Peruana Cayetano Heredia, Lima, Peru

8 Sociedad Científica de Estudiantes de Medicina Cayetano Heredia (SOCEMCH), Universidad Peruana Cayetano Heredia, Lima, Peru

9 Department of International Health, Bloomberg School of Public Health, Johns Hopkins University, Baltimore, MD USA

10 Nutrition and Dietetics, Universidad Científica del Sur. Lima, Perú

11 Hubert Department of Global Health, Rollins School of Public Health, Emory University, Atlanta, GA, USA

12 Emory Global Diabetes Research Center, Emory University, Atlanta, GA, USA

CONTENTS

- **Supplementary Figure 1.** Flowchart of the study population
- **Supplementary Figure 2.** Geographic distribution and patterns of vulnerable newborn phenotypes, Peru, 2016-2021.
- **Supplementary Figure 3.** Geographic distribution and patterns of vulnerable newborn phenotypes, 2012 – 2021
- **Supplementary Figure 4.** Temporal trends of six newborn phenotypes by regions, 2012 – 2021
- **Supplementary Figure 5.** Temporal trends of six newborn phenotypes among girls by regions, 2012 – 2021
- **Supplementary Figure 6.** Temporal trends of six newborn phenotypes among boys by regions, 2012 – 2021
- **Supplementary Figure 7.** Temporal trends of ten newborn phenotypes at national level, by sex and by natural regions, 2012 – 2021
- **Supplementary Figure 8.** Temporal trends of ten newborn phenotypes by regions, 2012 – 2021
- **Supplementary Figure 9.** Temporal trends of ten newborn phenotypes among girls by regions, 2012 – 2021
- **Supplementary Figure 10.** Temporal trends of ten newborn phenotypes among boys by regions, 2012 – 2021
- **Supplementary Figure 11.** Equiplots of vulnerable newborn phenotypes in younger and less educated mothers, 2015-2021
- Notes: The size of the bubbles is proportional to the number of live births.
- **Supplementary Figure 12.** Socioeconomic and geographic inequities of ten vulnerable newborn phenotypes in terms of maternal education, 2012 – 2021
- **Supplementary Figure 13.** Socioeconomic and geographic inequities of ten vulnerable newborn phenotypes in terms of maternal age, 2012 – 2021
- **Supplementary Figure 14.** Socioeconomic and geographic inequities of ten vulnerable newborn phenotypes in terms of healthcare provider, 2012 – 2021
- **Supplementary Figure 15.** Socioeconomic and geographic inequities of ten vulnerable newborn phenotypes in terms of altitude, 2012 – 2021
- **Supplementary Table 1.** Strengthening the Reporting of Observational studies in Epidemiology (STROBE) checklist

Supplementary Figure 1. Flowchart of the study population

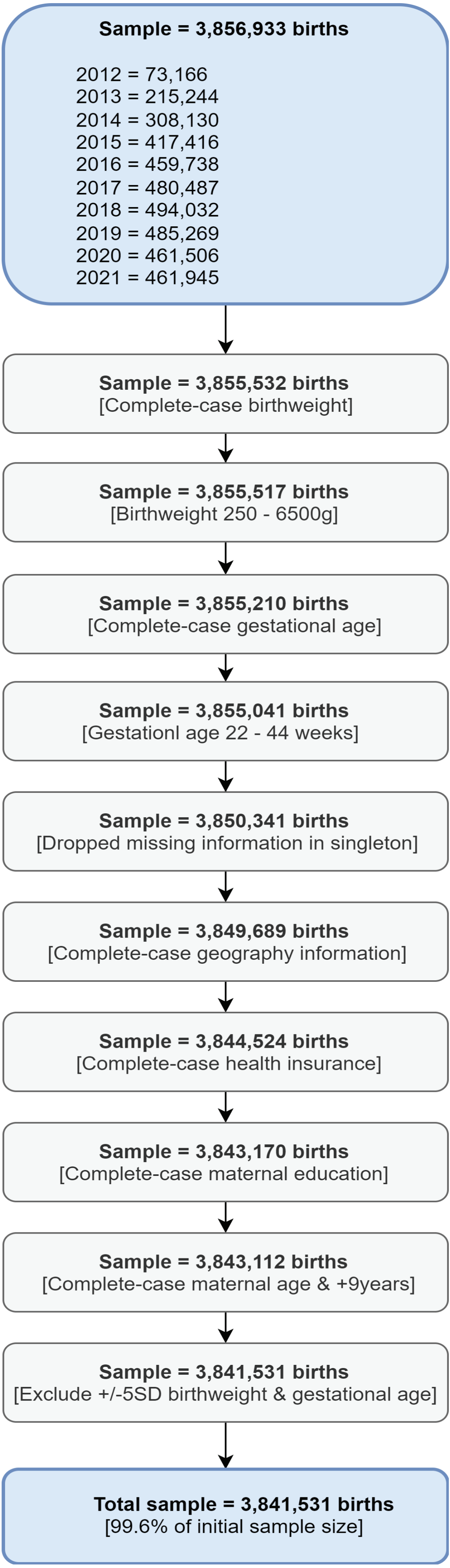

Supplementary Figure 2. Geographic distribution and patterns of vulnerable newborn phenotypes, 2016-2021

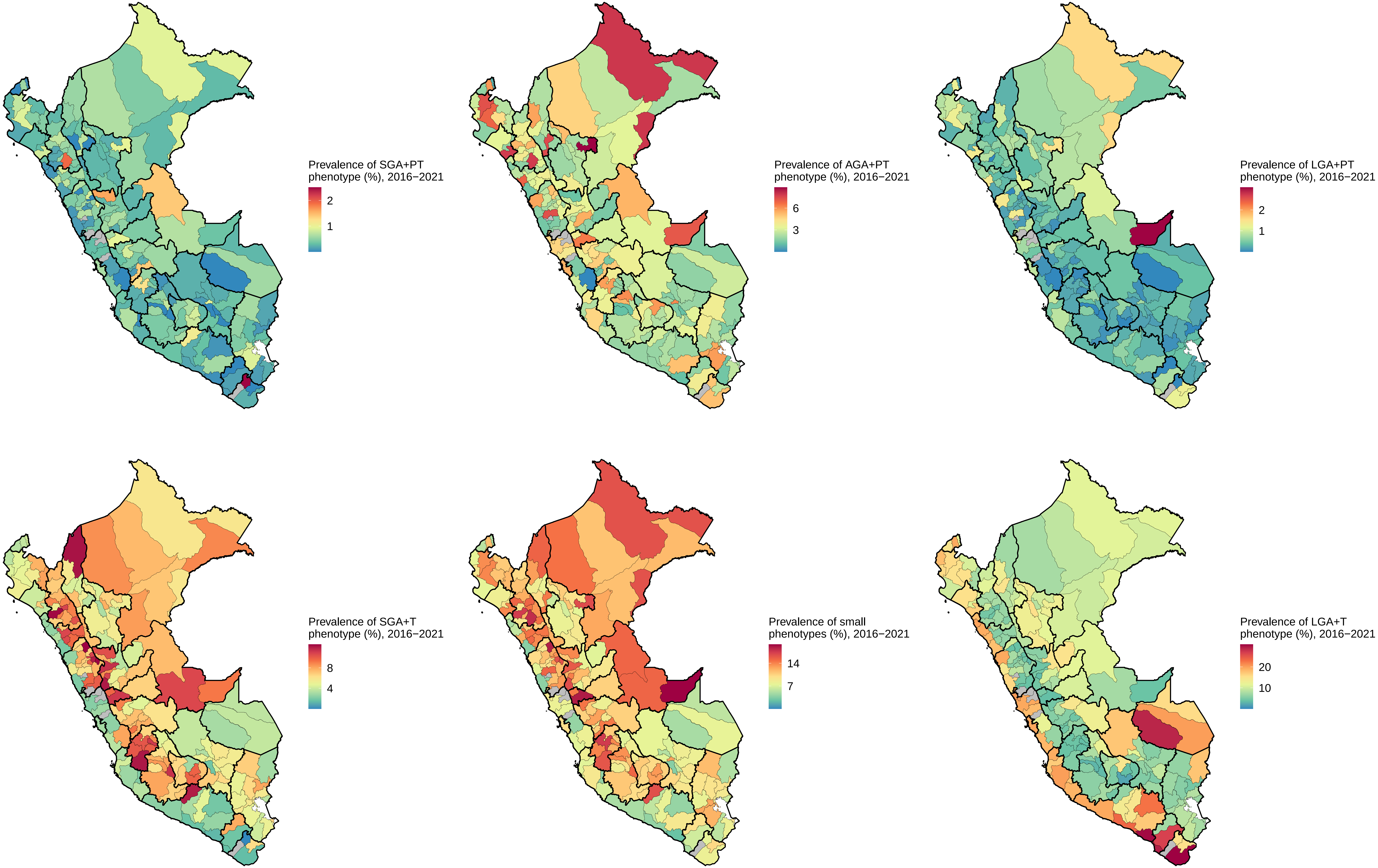

Supplementary Figure 3. Geographic distribution and patterns of nine vulnerable newborn phenotypes, 2012-2021

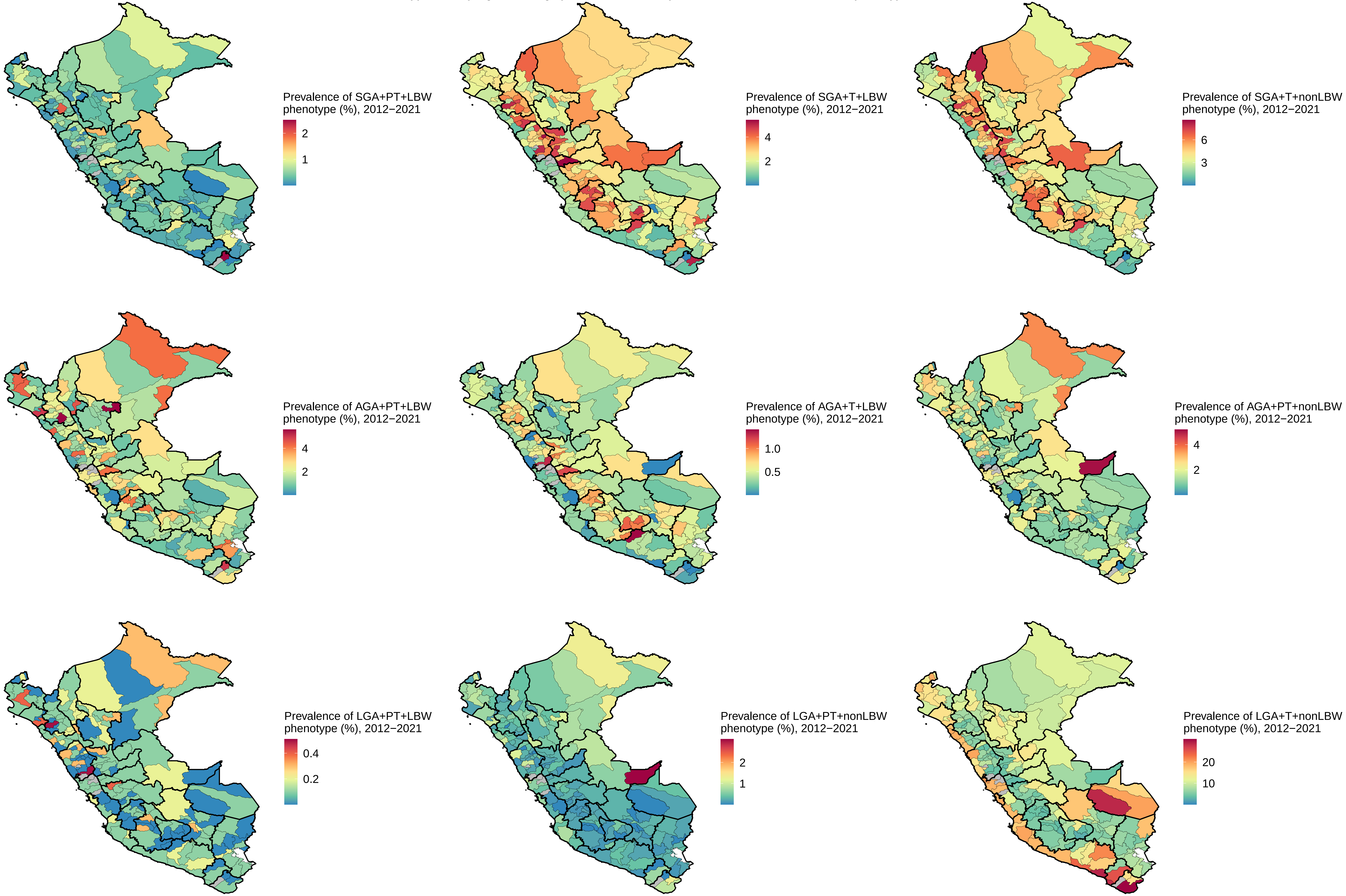

Supplementary Figure 4. Temporal trends of six newborn phenotypes by regions, 2012 - 2021

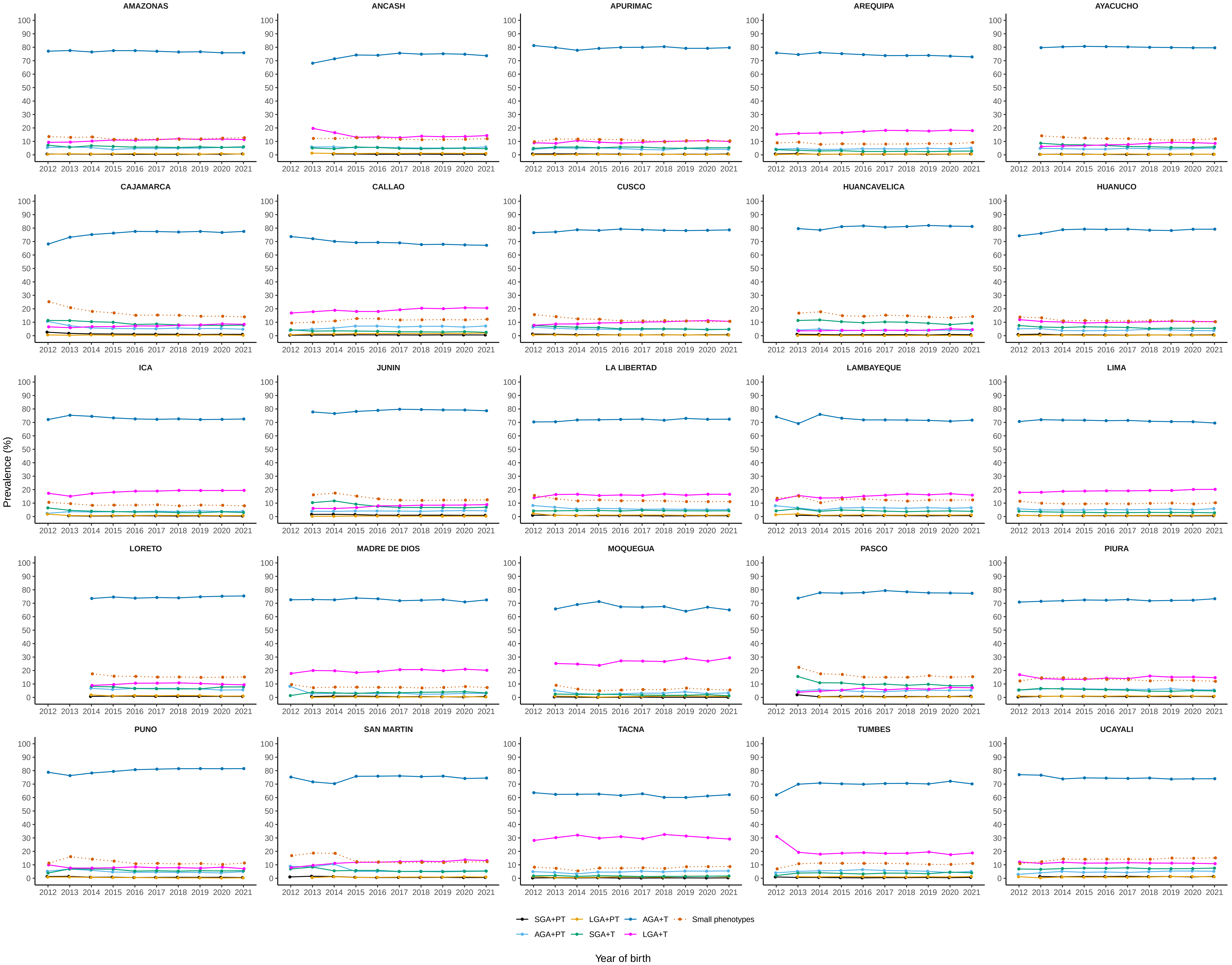

Supplementary figure 5. Temporal trends of six newborn phenotypes among girls by regions, 2012 - 2021

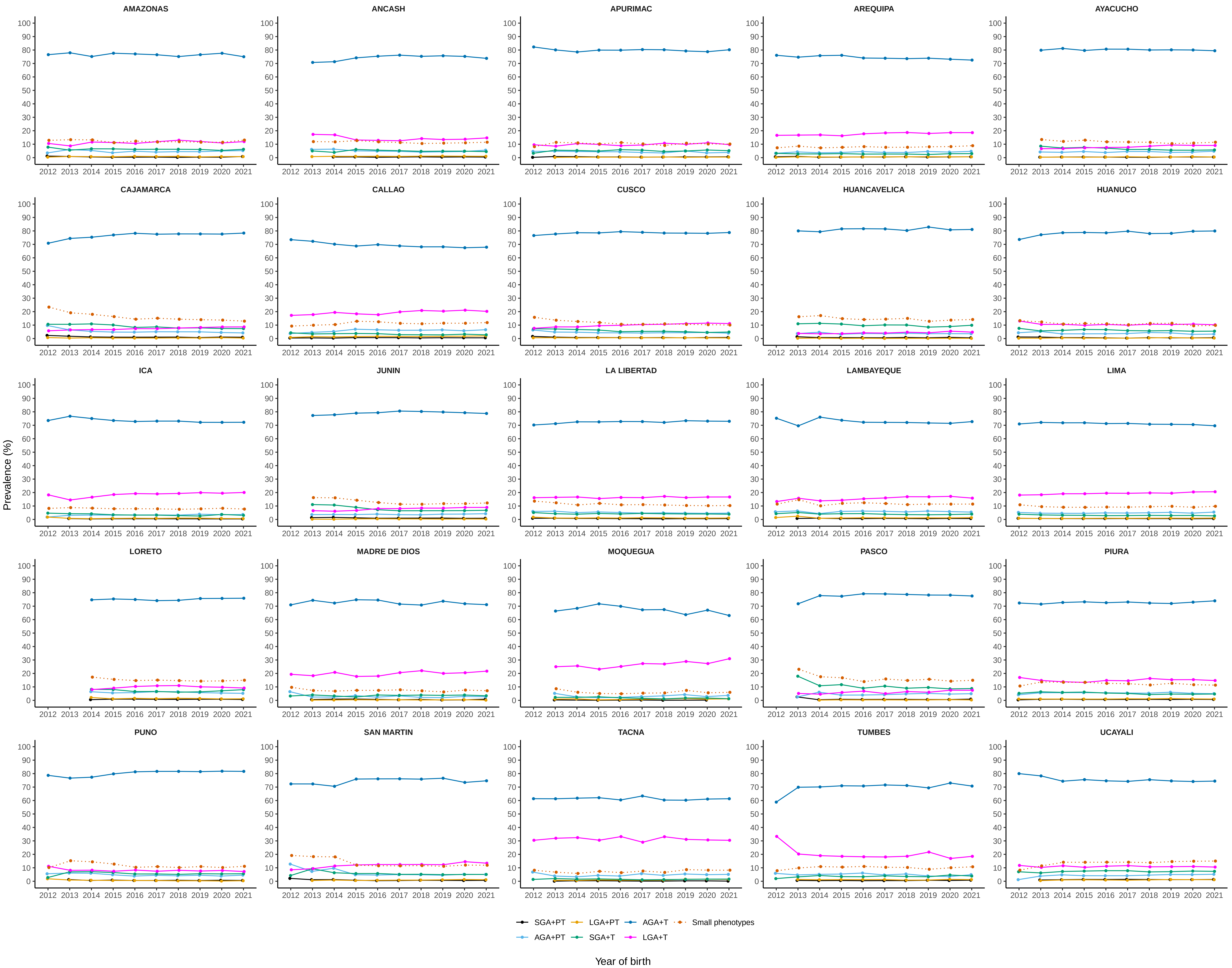

Supplementary Figure 6. Temporal trends of six newborn phenotypes among boys by regions, 2012 - 2021

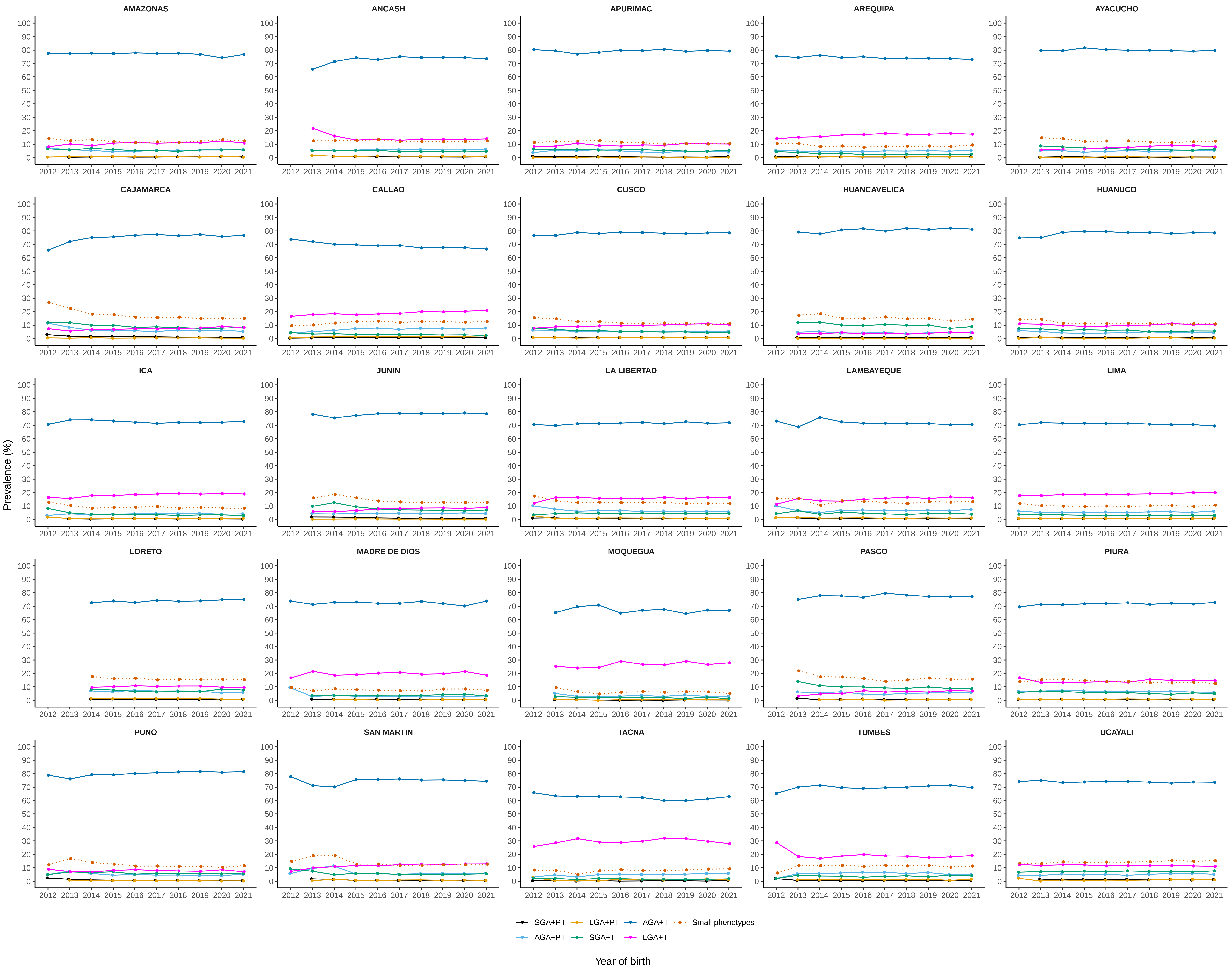

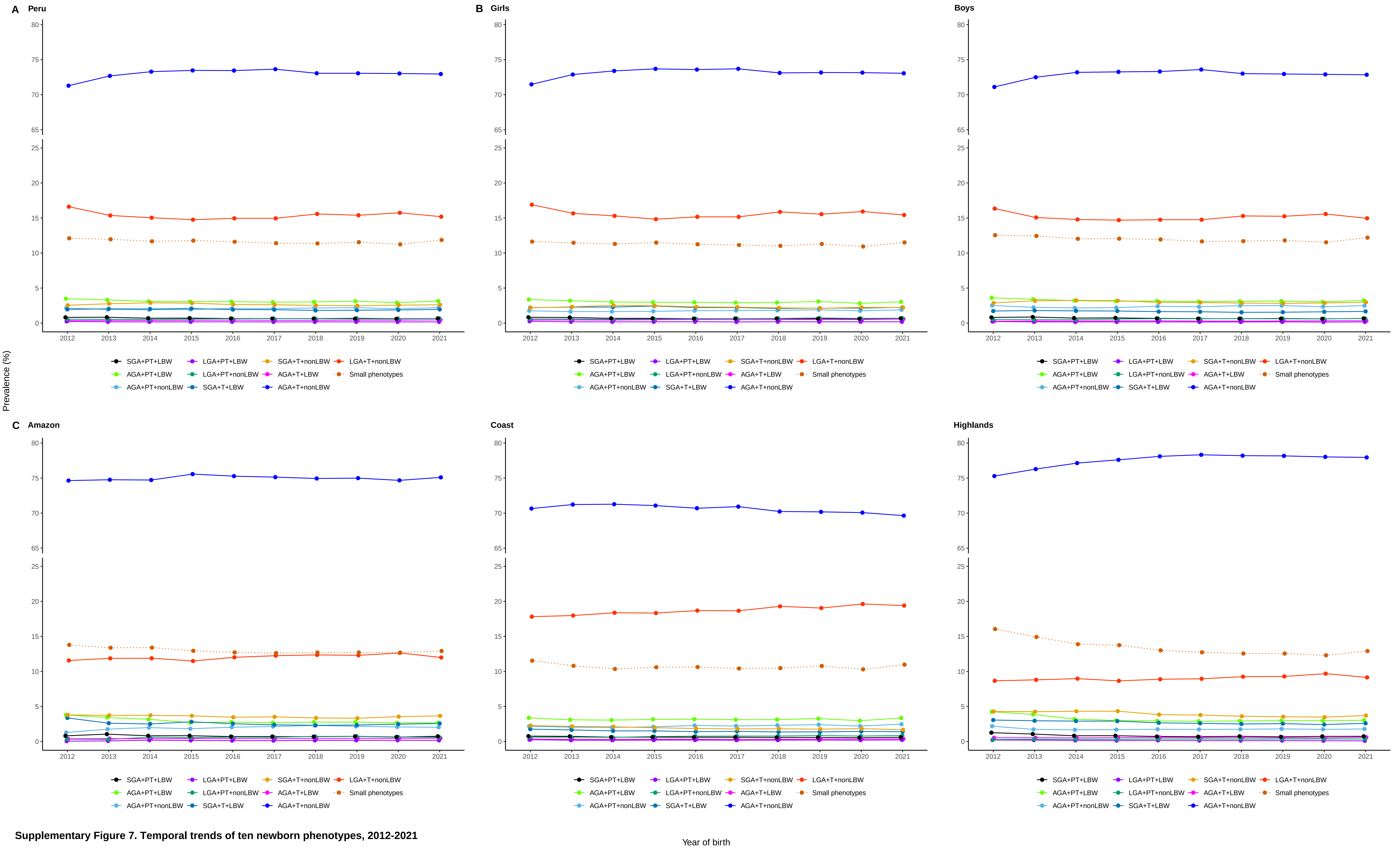

Supplementary Figure 7. Temporal trends of ten newborn phenotypes, 2012-2021

Supplementary Figure 8. Temporal trends of ten newborn phenotypes by regions, 2012 - 2021

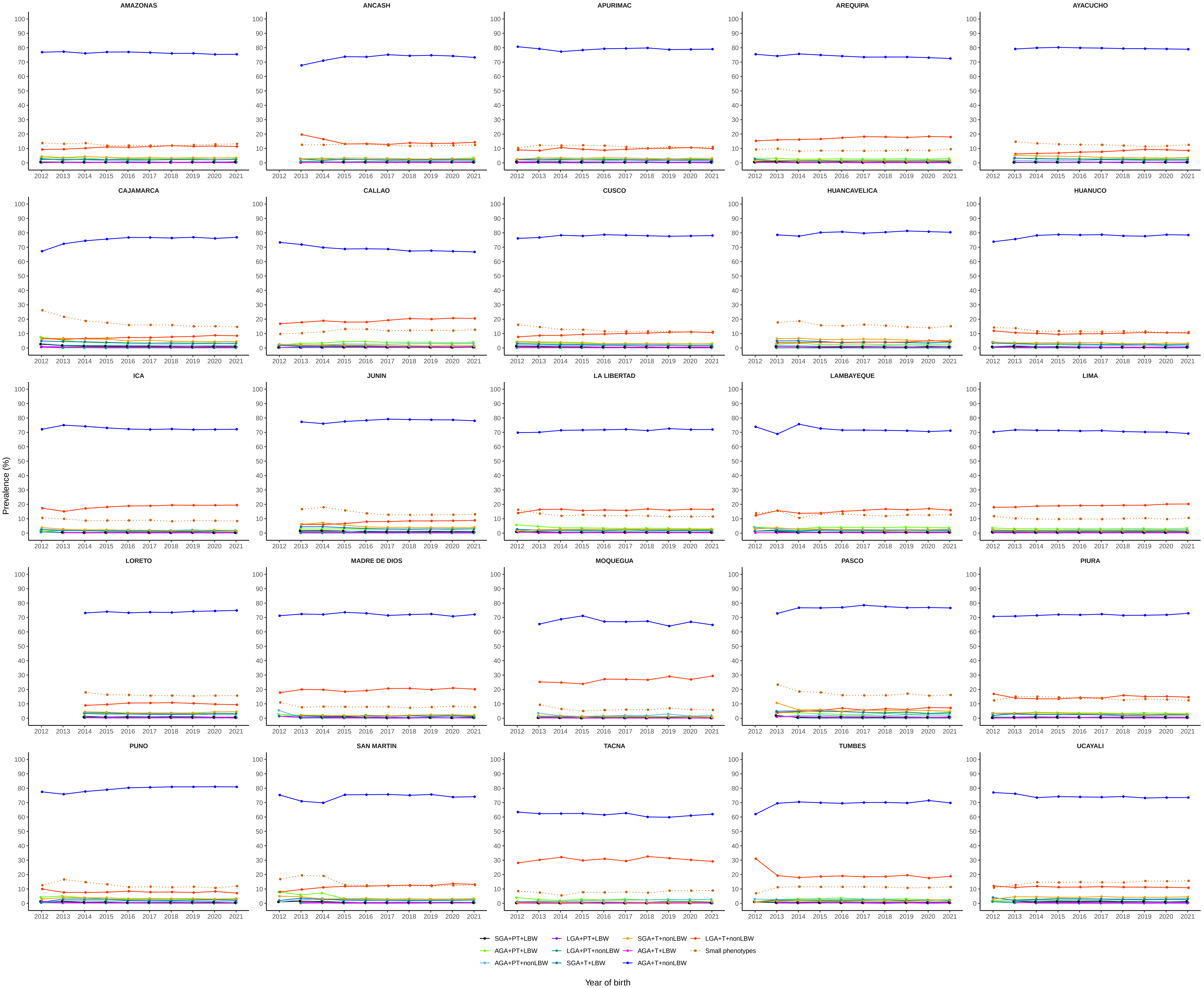

Supplementary Figure 9. Temporal trends of newborn phenotypes among girls by regions, 2012 - 2021

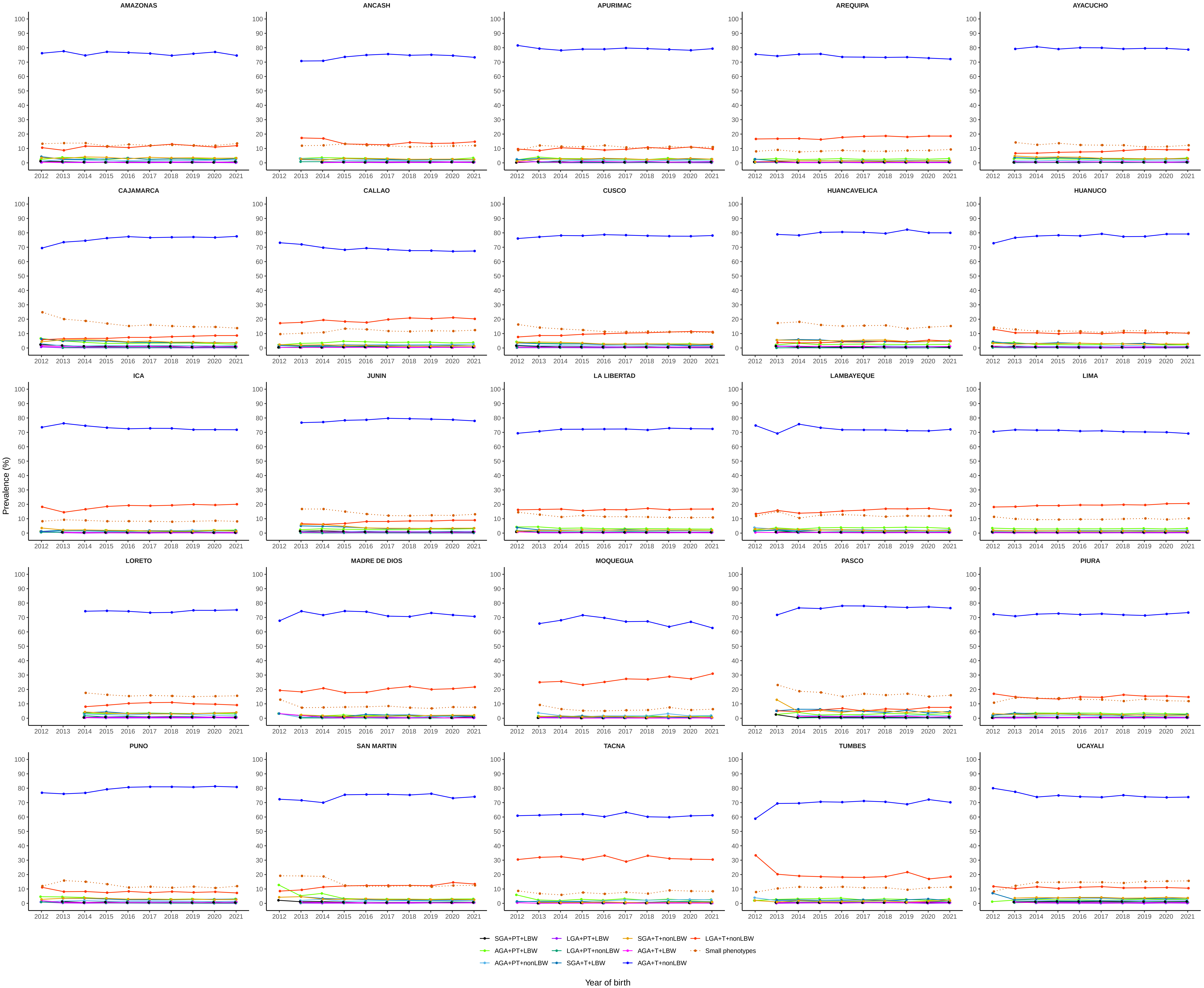

Supplementary Figure 10. Temporal trends of ten newborn phenotypes among boys by regions, 2012 - 2021

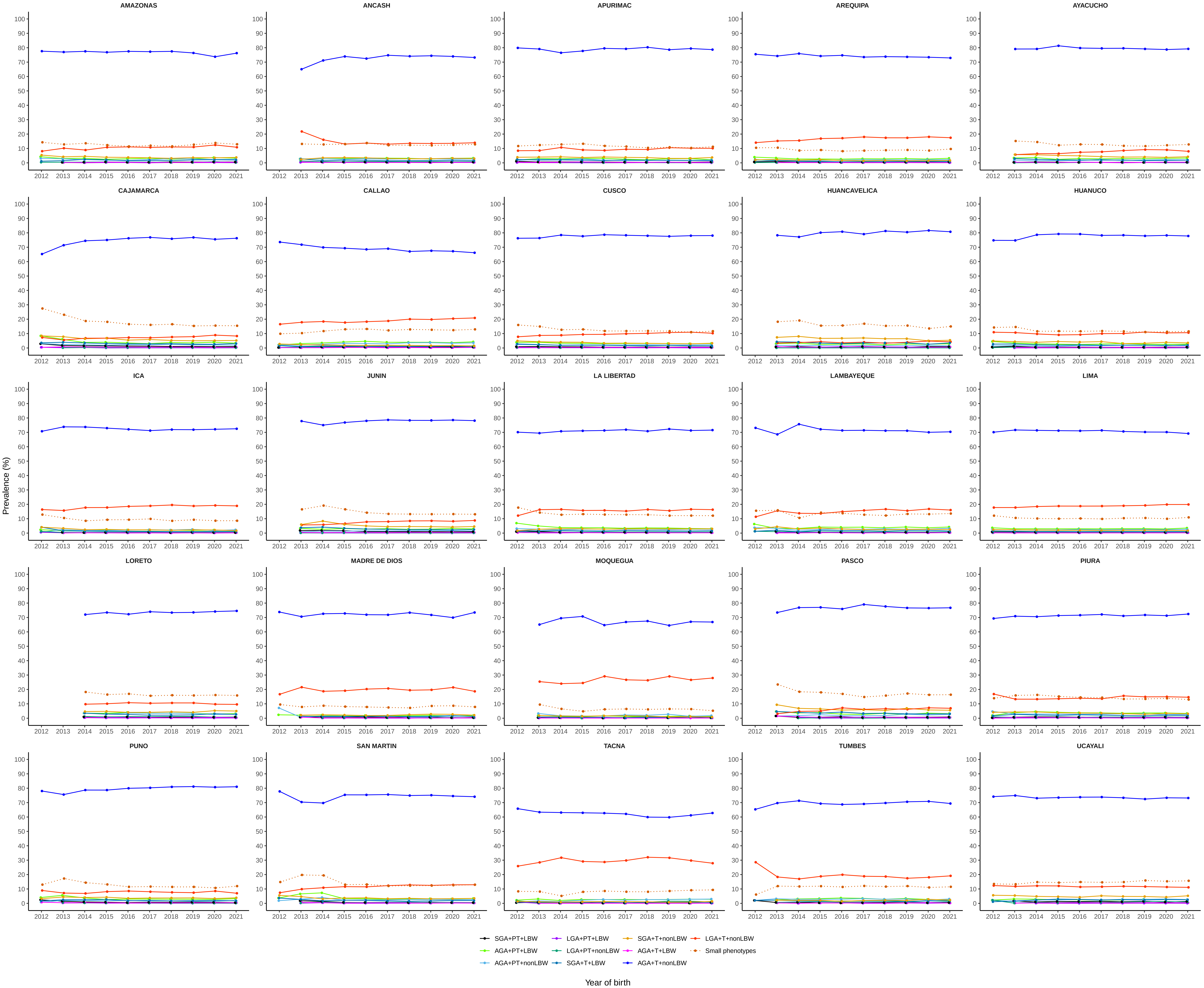

Supplementary Figure 11. Equiplots of vulnerable newborn phenotypes, 2015 - 2021

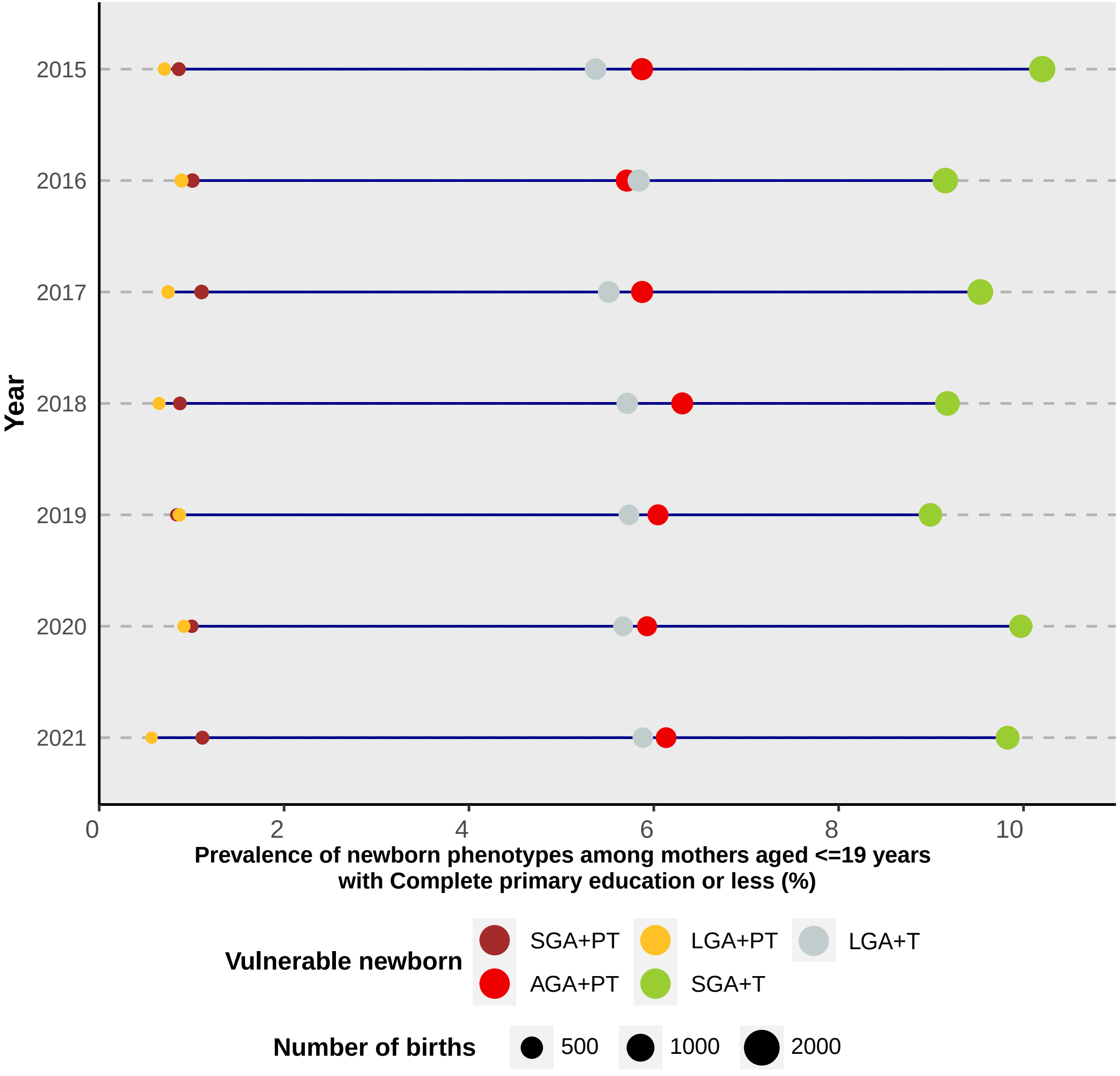

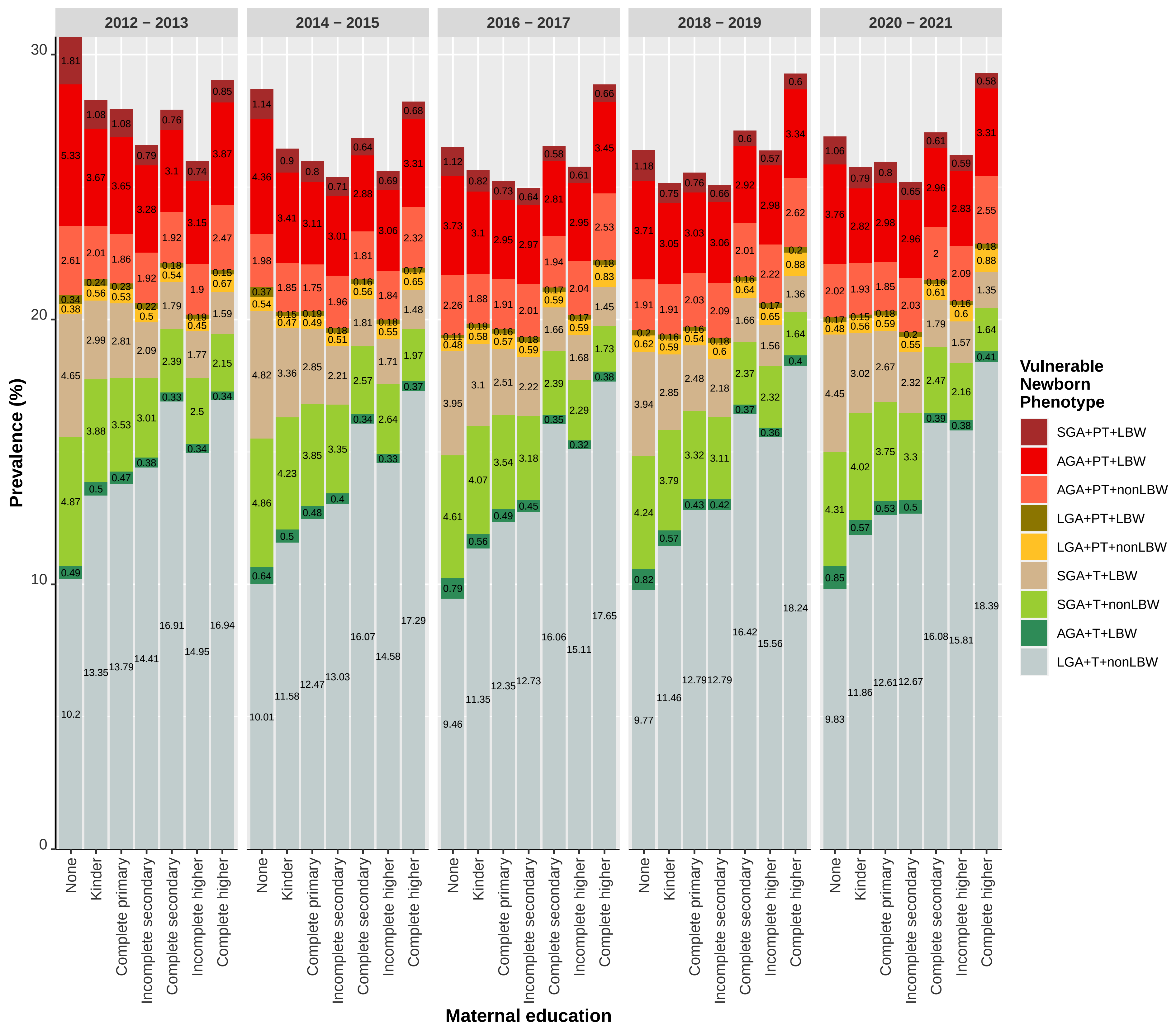
2012 – 2013
2014 – 2015
2016 – 2017
2018 – 2019
2020 – 2021
Prevalence (%)
0
10
20
30
None
Kinder
Complete primary
Incomplete secondary
Complete secondary
Incomplete higher
Complete higher
Maternal education
Vulnerable Newborn Phenotype
SGA+PT+LBW
AGA+PT+LBW
AGA+PT+nonLBW
LGA+PT+LBW
LGA+PT+nonLBW
SGA+T+LBW
SGA+T+nonLBW
AGA+T+LBW
LGA+T+nonLBW

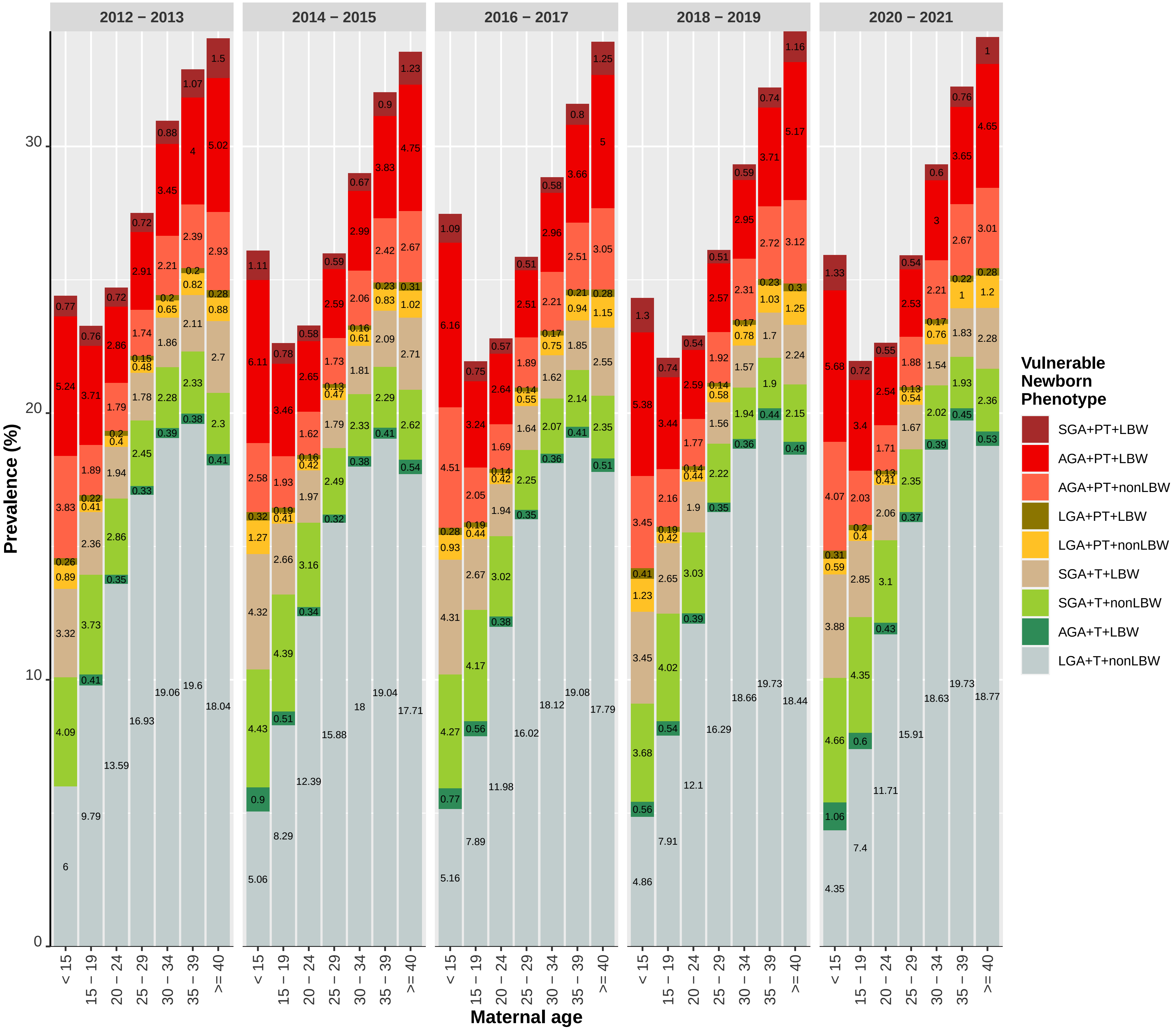

2012 − 2013
2014 − 2015
2016 − 2017
2018 − 2019
2020 − 2021
Prevalence (%)
Maternal age
< 15
15 − 19
20 − 24
25 − 29
30 − 34
35 − 39
>= 40
Vulnerable Newborn Phenotype
SGA+PT+LBW
AGA+PT+LBW
AGA+PT+nonLBW
LGA+PT+LBW
LGA+PT+nonLBW
SGA+T+LBW
SGA+T+nonLBW
AGA+T+LBW
LGA+T+nonLBW

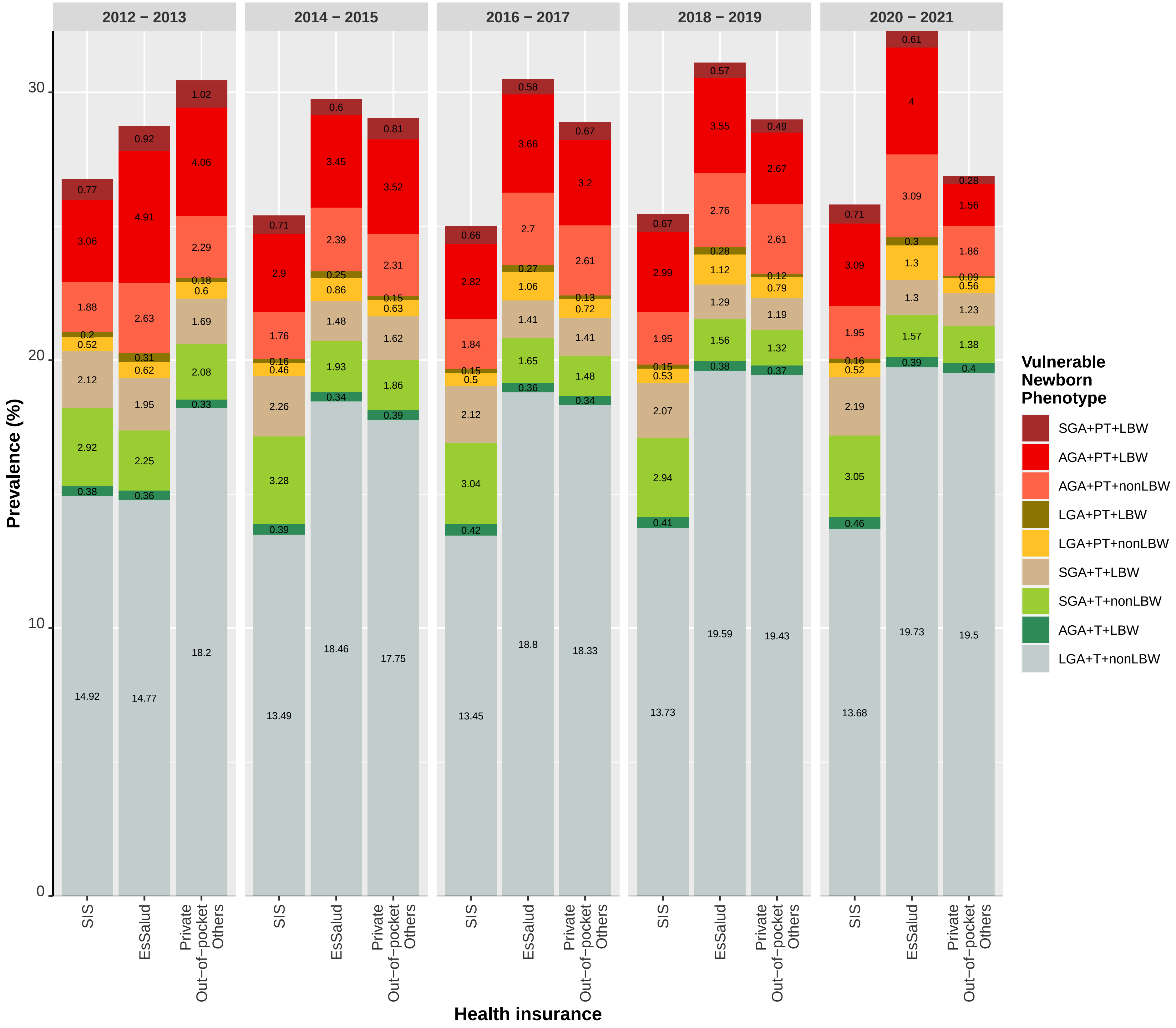
2012 – 2013
2014 – 2015
2016 – 2017
2018 – 2019
2020 – 2021
Prevalence (%)
0
10
20
30
0.77
3.06
1.88
0.2
0.52
2.12
2.92
0.38
14.92
0.92
4.91
2.63
0.31
0.62
1.95
2.25
0.36
14.77
1.02
4.06
2.29
0.18
0.6
1.69
2.08
0.33
18.2
0.71
2.9
1.76
0.16
0.46
2.26
3.28
0.39
13.49
0.6
3.45
2.39
0.25
0.86
1.48
1.93
0.34
18.46
0.81
3.52
2.31
0.15
0.63
1.62
1.86
0.39
17.75
0.66
2.82
1.84
0.15
0.5
2.12
3.04
0.42
13.45
0.58
3.66
2.7
0.27
1.06
1.41
1.65
0.36
18.8
0.67
3.2
2.61
0.13
0.72
1.41
1.48
0.34
18.33
0.67
2.99
1.95
0.15
0.53
2.07
2.94
0.41
13.73
0.57
3.55
2.76
0.28
1.12
1.29
1.56
0.38
19.59
0.49
2.67
2.61
0.12
0.79
1.19
1.32
0.37
19.43
0.71
3.09
1.95
0.16
0.52
2.19
3.05
0.46
13.68
0.61
4
3.09
0.3
1.3
1.3
1.57
0.39
19.73
0.28
1.56
1.86
0.09
0.56
1.23
1.38
0.4
19.5
SIS
EsSalud
Private
Out−of−pocket
Others
Health insurance
Vulnerable Newborn Phenotype
SGA+PT+LBW
AGA+PT+LBW
AGA+PT+nonLBW
LGA+PT+LBW
LGA+PT+nonLBW
SGA+T+LBW
SGA+T+nonLBW
AGA+T+LBW
LGA+T+nonLBW

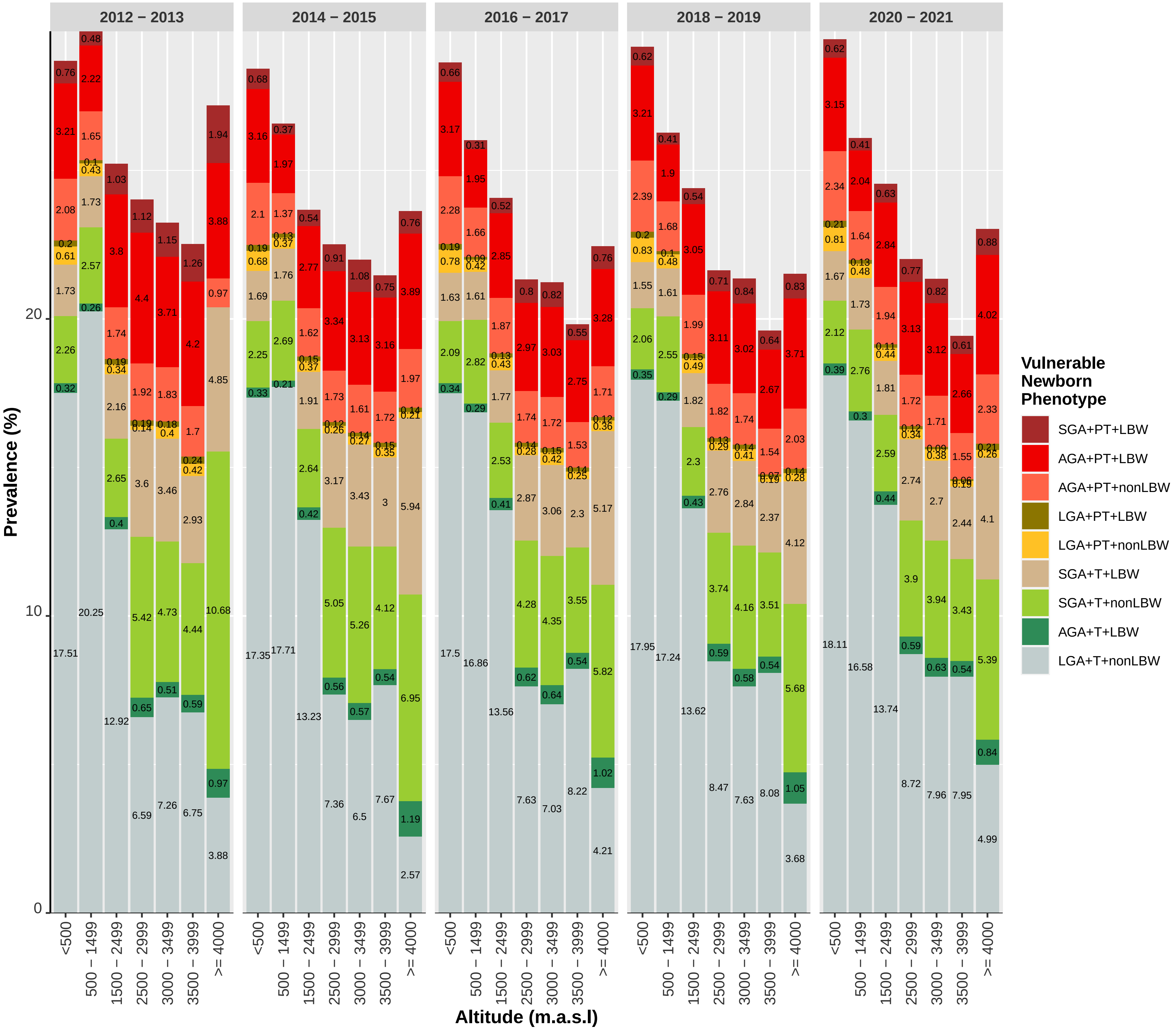
2012 – 2013
2014 – 2015
2016 – 2017
2018 – 2019
2020 – 2021
Prevalence (%)
0
10
20
<500
500 – 1499
1500 – 2499
2500 – 2999
3000 – 3499
3500 – 3999
>= 4000
Altitude (m.a.s.l)
Vulnerable Newborn Phenotype
SGA+PT+LBW
AGA+PT+LBW
AGA+PT+nonLBW
LGA+PT+LBW
LGA+PT+nonLBW
SGA+T+LBW
SGA+T+nonLBW
AGA+T+LBW
LGA+T+nonLBW

Supplementary table 1.

Strengthening the Reporting of Observational studies in Epidemiology (STROBE) checklist

STROBE Statement—checklist of items that should be included in reports of observational studies

	Item No	Recommendation	Page
**Title and abstract**	1	*(a)* Indicate the study's design with a commonly used term in the title or the abstract<br>*(b)* Provide in the abstract an informative and balanced summary of what was done and what was found	1,4
**Introduction**			
Background/rationale	2	Explain the scientific background and rationale for the investigation being reported	7-8
Objectives	3	State specific objectives, including any prespecified hypotheses	8
**Methods**			
Study design	4	Present key elements of study design early in the paper	9
Setting	5	Describe the setting, locations, and relevant dates, including periods of recruitment, exposure, follow-up, and data collection	9
Participants	6	*(a)* Give the eligibility criteria, and the sources and methods of selection of participants. Describe methods of follow-up<br>*(b)* For matched studies, give matching criteria and number of exposed and unexposed	9
Variables	7	Clearly define all outcomes, exposures, predictors, potential confounders, and effect modifiers. Give diagnostic criteria, if applicable	10
Data sources/ measurement	8*	For each variable of interest, give sources of data and details of methods of assessment (measurement). Describe comparability of assessment methods if there is more than one group	8
Bias	9	Describe any efforts to address potential sources of bias	11,15-17
Study size	10	Explain how the study size was arrived at	9
Quantitative variables	11	Explain how quantitative variables were handled in the analyses. If applicable, describe which groupings were chosen and why	10
Statistical methods	12	*(a)* Describe all statistical methods, including those used to control for confounding<br>*(b)* Describe any methods used to examine subgroups and interactions<br>*(c)* Explain how missing data were addressed<br>*(d)* If applicable, explain how loss to follow-up was addressed<br>*(e)* Describe any sensitivity analyses	10-11

Continued on next page

Results			Page
Participants	13*	(a) Report numbers of individuals at each stage of study—e.g., numbers potentially eligible, examined for eligibility, confirmed eligible, included in the study, completing follow-up, and analyzed<br>(b) Give reasons for non-participation at each stage	9
		© Consider use of a flow diagram	Supplementary Figure 1
Descriptive data	14*	(a) Give characteristics of study participants (e.g., demographic, clinical, social) and information on exposures and potential confounders<br>(b) Indicate number of participants with missing data for each variable of interest<br>(c) Summarize follow-up time (e.g., average and total amount)	9
Outcome data	15*	Report numbers of outcome events or summary measures over time	10, 12
Main results	16	(a) Give unadjusted estimates and, if applicable, confounder-adjusted estimates and their precision (e.g., 95% confidence interval). Make clear which confounders were adjusted for and why they were included<br>(b) Report category boundaries when continuous variables were categorized<br>(c) If relevant, consider translating estimates of relative risk into absolute risk for a meaningful time period	12-15/Tables & Figures
Other analyses	17	Report other analyses done—e.g., analyses of subgroups and interactions, and sensitivity analyses	Supplementary Figures &Tables
**Discussion**			
Key results	18	Summarize key results with reference to study objectives	15
Limitations	19	Discuss limitations of the study, taking into account sources of potential bias or imprecision.<br>Discuss both direction and magnitude of any potential bias	15-17
Interpretation	20	Give a cautious overall interpretation of results considering objectives, limitations, multiplicity of analyses, results from similar studies, and other relevant evidence	17-19
Generalizability	21	Discuss the generalizability (external validity) of the study results	15-17
**Other information**			
Funding	22	Give the source of funding and the role of the funders for the present study and, if applicable, for the original study on which the present article is based	2,4,11

*Give information separately for cases and controls in case-control studies and, if applicable, for exposed and unexposed groups in cohort and cross-sectional studies.

Note: An Explanation and Elaboration article discusses each checklist item and gives methodological background and published examples of transparent reporting. The STROBE checklist is best used in conjunction with this article (freely available on the Web sites of PLoS Medicine at http://www.plosmedicine.org/, Annals of Internal Medicine at http://www.annals.org/, and Epidemiology at http://www.epidem.com/). Information on the STROBE Initiative is available at www.strobe-statement.org.
